# Are genetic databases sufficiently populated to detect non-indigenous species?

**DOI:** 10.1007/s10530-016-1134-1

**Published:** 2016-04-05

**Authors:** Elizabeta Briski, Sara Ghabooli, Sarah A. Bailey, Hugh J. MacIsaac

**Affiliations:** 1 GEOMAR, Helmholtz-Zentrum für Ozeanforschung Kiel, 24105 Kiel, Germany; 2 Great Lakes Institute for Environmental Research, University of Windsor, Windsor, ON N9B 3P4 Canada; 3 Great Lakes Laboratory for Fisheries and Aquatic Sciences, Fisheries and Oceans Canada, Burlington, ON L7S 1A1 Canada

**Keywords:** Aquatic taxa, Biological invasion, DNA barcoding, Molecular databases, Species identification, Terrestrial taxa

## Abstract

Correct species identifications are of tremendous importance for invasion ecology, as mistakes could lead to misdirecting limited resources against harmless species or inaction against problematic ones. DNA barcoding is becoming a promising and reliable tool for species identifications, however the efficacy of such molecular taxonomy depends on gene region(s) that provide a unique sequence to differentiate among species and on availability of reference sequences in existing genetic databases. Here, we assembled a list of aquatic and terrestrial non-indigenous species (NIS) and checked two leading genetic databases for corresponding sequences of six genome regions used for DNA barcoding. The genetic databases were checked in 2010, 2012, and 2016. All four aquatic kingdoms (Animalia, Chromista, Plantae and Protozoa) were initially equally represented in the genetic databases, with 64, 65, 69, and 61 % of NIS included, respectively. Sequences for terrestrial NIS were present at rates of 58 and 78 % for Animalia and Plantae, respectively. Six years later, the number of sequences for aquatic NIS increased to 75, 75, 74, and 63 % respectively, while those for terrestrial NIS increased to 74 and 88 % respectively. Genetic databases are marginally better populated with sequences of terrestrial NIS of plants compared to aquatic NIS and terrestrial NIS of animals. The rate at which sequences are added to databases is not equal among taxa. Though some groups of NIS are not detectable at all based on available data—mostly aquatic ones—encouragingly, current availability of sequences of taxa with environmental and/or economic impact is relatively good and continues to increase with time.

**Electronic supplementary material:**

The online version of this article (doi:10.1007/s10530-016-1134-1) contains supplementary material, which is available to authorized users.

## Introduction

Biological invasions are a complex process that can be viewed as a series of stages, including transport, introduction, establishment and spread (Kolar and Lodge 2001; Colautti and MacIsaac 2004). Management efforts focused on interrupting the invasion process, particularly at the transport or introduction stage, are of great significance as they are more effective than eradication or control of established populations of non-indigenous species (NIS) (Lodge et al. 2006; Lockwood et al. 2007; Hulme et al. 2008). Many transport vectors, however, are still not effectively managed, and species continue to arrive in new habitats (Hulme et al. 2008; Kelly et al. 2009; Conn et al. 2010; Sephton et al. 2011; Briski et al. 2012a, b, 2013). Additionally, incomplete taxonomic, biogeographic and historical data frequently result in an inability to determine if newly reported species are native or non-indigenous (Carlton 2009). Incorrect species identifications could artificially inflate or depress the number of NIS in an ecosystem, and lead to misdirecting limited resources against harmless species or inaction against problematic ones (Bax et al. 2001; Simberloff 2009). As a result, accurate identification of species is typically highlighted as an essential component of invasion management strategies (Bax et al. 2001).

DNA barcoding is becoming a promising and reliable tool for species identifications (Cross et al. 2010; Briski et al. 2011). Particularly in invasion ecology, where early detection is tremendously important, molecular identification has several advantages over morphological identification (Cross et al. 2010; Briski et al. 2011). The latter often requires examination of mature specimens of a particular sex, or flowering or fruiting specimens for some plant species (Radford et al. 1968; Cross et al. 2010), which may or may not be present in initial collections of individuals from a new habitat. In contrast, molecular methods allow identification of NIS at any life stage, based on successful DNA extraction from a single individual, egg, or seed—possibly facilitating early detection of NIS before an introduced population becomes fully established in an area (Armstrong and Bell 2005; Chown et al. 2008; Briski et al. 2011; Zhan and MacIsaac 2015). Early identification of NIS, followed by immediate eradication before reproductive or flowering phases, may prevent distribution of eggs, seeds or pollen, circumventing the establishment of the next generation, admixture of genetic material among distinct NIS populations or hybridization with closely related species (Kolbe et al. 2007; Ayres et al. 2008; Cross et al. 2010). Furthermore, new sequencing technologies, collectively called “Next-Generation Sequencing”, have the ability to generate massive amounts of sequence data in one run and allow screening of whole ecosystems (Hall 2007; Rokas and Abbot 2009; Zhan et al. 2013; Zhan and MacIsaac 2015). By assessing multiple barcoding regions using universal primers, it is possible to simultaneously identify not only NIS, but also their associated microbiota, parasites and fellow travelers (Cross et al. 2010).

Use of DNA barcodes for species identification has its own weaknesses. The efficacy of DNA barcoding depends on gene region/s that provide a unique sequence to differentiate among species (Hebert et al. 2003; Cross et al. 2010) and availability of reference sequences in existing genetic databases (Darling and Blum 2007; Briski et al. 2011). Originally, the aim was to have one DNA barcode that would discriminate among all species across all phyla (Janzen 2004; Hebert and Gregory 2005), but this objective has proven unlikely as genomes vary considerably (Shearer and Coffroth 2008; Cross et al. 2010). Consequently, the cytochrome *c* oxidase subunit I (COI) gene has become the standard DNA barcoding marker for most animal groups (Hebert et al. 2003), the internal transcribed spacer (ITS) has been applied for a wide array of groups including plants, fungi, algae, and animals (Kress et al. 2005), while ribulose-bisphosphate carboxylase (rbcL) and maturase K (matK) genes differentiate most plants (Hollingsworth et al. 2009). The availability of reference sequences in genetic databases for these gene regions varies among taxonomic groups (Briski et al. 2011). We recently reported that only 5, 3.5, and 3.5 % of all described Rotifera, Bryozoa, and Copepoda species, respectively, had reference sequences of COI or small subunit ribosomal 16S rDNA (16S) in the Barcode of Life Database (BOLD) or GenBank (Briski et al. 2011); however, 54 % of known Branchiopoda species are represented. The Consortium for the Barcode of Life fosters development of international alliances to build a global barcode library, continuously increasing the number of available species barcode sequences in the BOLD database to create a global bio-identification system covering all eukaryotic taxa (Ratnasingham and Hebert 2007). In contrast, GenBank was designed to provide access within the scientific community to the most up-to-date and comprehensive DNA sequence information. GenBank is not restricted to specific regions of the genome, and includes sequences developed for a variety of research purposes (NCBI 2015). Consequently, taxa studied, for example for medicine, pharmacy, or model species in ecological and evolutionary studies, may be better represented in GenBank.

Considering the importance of rapid identification of newly reported species in an area, and noting the different goals and applications of the two aforementioned genetic databases, this study explored availability of DNA sequences for identification of NIS. We assembled a global list of aquatic and terrestrial NIS, and then searched these databases for six genome regions relevant for species-level identification to determine the potential utility of molecular methods in invasion management. To check for an enrichment trend in the genetic databases, the databases were searched three times, in summer 2010 and 2012, and in January 2016.

## Methods

From May to September 2010 we utilized Thomson’s Institute for Science Information (ISI) Web of Knowledge 4.0 to search the scientific literature to assemble a global list of aquatic and terrestrial NIS. Initially, the following search terms were used: non-native OR alien OR exotic OR non-indigenous OR introduced OR colonizing—resulting in 29,975 publications. Our results were narrowed with an additional search term: list—which also improved the prevalence of studies reporting species newly reported in a region and reduced the importance of well-studied high impact NIS (Pyšek et al. 2008). The resulting 436 publications were screened for NIS reports, and 55 were used to assemble our global list (Appendix 1 of ESM). In addition to NIS recovered by Thomson’s ISI search, we included species listed in the Global Invasive Species Database of the Invasive Species Specialist Group (ISSG 2010). To reduce geographical bias, we did not include species from regional data sets such as Delivering Alien Invasive Species Inventories for Europe (DAISIE) or Great Lakes Aquatic Nonindigenous Information System (GLANSIS) (Pyšek et al. 2008). Bacteria, virus-like particles and fungi were excluded from our list because these taxa typically have uncertain status as non-indigenous or native. After the list was assembled, the recorded species were assigned to kingdom, phylum, and class by consulting several taxonomic websites [e.g. BOLD, the European Nature Information System (EUNIS), World Register of Marine Species (WORMS), ZipcodeZoo].

To determine the potential for molecular identification of NIS, we searched BOLD (http://www.boldsystems.org/) and GenBank (http://www.ncbi.nlm.nih.gov/genbank/) for COI, 16S, small subunit ribosomal 18S rDNA (18S), ITS, rbcL and matK gene sequences. To examine the incidence of sequence deposition to genetic databases, we assessed both genetic databases three times: from May to September 2010, from June to August 2012, and in January 2016. In 2010 and 2012, BOLD was assessed only for COI sequences as in these years it contained very few ITS, rbcL or matK, and no 16S or 18S sequences; in 2016, it was assessed for all six genome regions. GenBank was assessed for all six genome regions each time. To determine the rate of sequence deposition to genetic databases, a series of regression analyses were conducted with total number of species with at least one sequence in at least one genetic database as the dependent variables and time as the independent variable. Additionally, to compare the trend of deposition of sequences of NIS on our list to general deposition of sequences to BOLD irrespective of indigenous/non-indigenous status, regression analysis for BOLD with all species in BOLD with at least one sequence as the dependent variable and time as the independent variable was conducted as well (consulted 17 February 2016).

Finally, to explore if some classes (hereafter class/es is used in the systematic sense) of NIS were more or less represented in genetic databases than was the average for taxa within its particular habitat (i.e. aquatic or terrestrial) in the years we examined (i.e. 2010, 2012, and 2016), we constructed scatter plots with number of NIS per class on the x-axis and number of NIS with at least one sequence in at least one genetic database per class on the y-axis; the line of unity was based on the average percentage of NIS with at least one sequence in at least one genetic database. Six different scatter plots and lines of unity were constructed: for aquatic taxa in 2010, 2012, and 2016, and for terrestrial taxa in 2010, 2012, and 2016. Values were log transformed to standardize the data. Primary dataset containing the list of aquatic and terrestrial NIS, their taxonomic determination, and availability of sequences in 2010, 2012 and 2016 is available at: doi:10.1594/PANGAEA.859211.

## Results

### Aquatic and terrestrial NIS

**Fig. 1:**
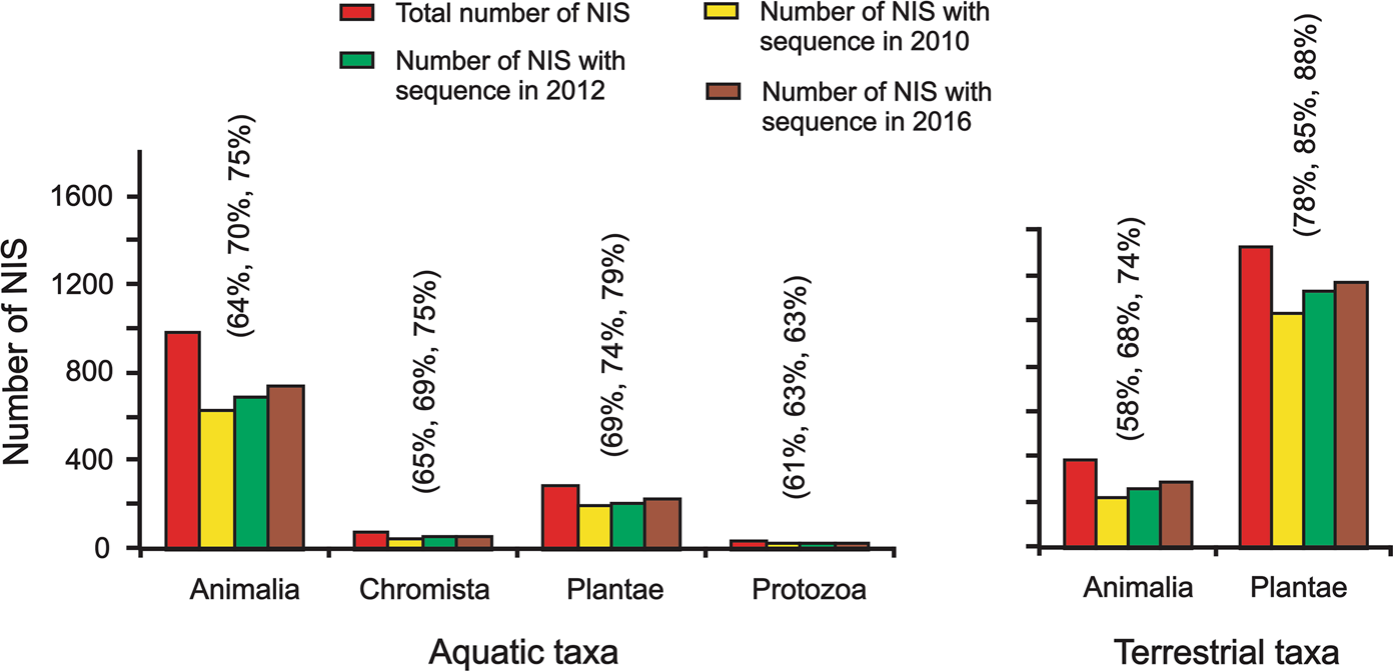
Number of non-indigenous species (NIS) per kingdom, and number of NIS with at least one sequence in at least one genetic database in 2010, 2012 and 2016 for aquatic and terrestrial taxa. Percentage cover for 2010, 2012 and 2016 are shown in *brackets*, respectively

**Fig. 2:**
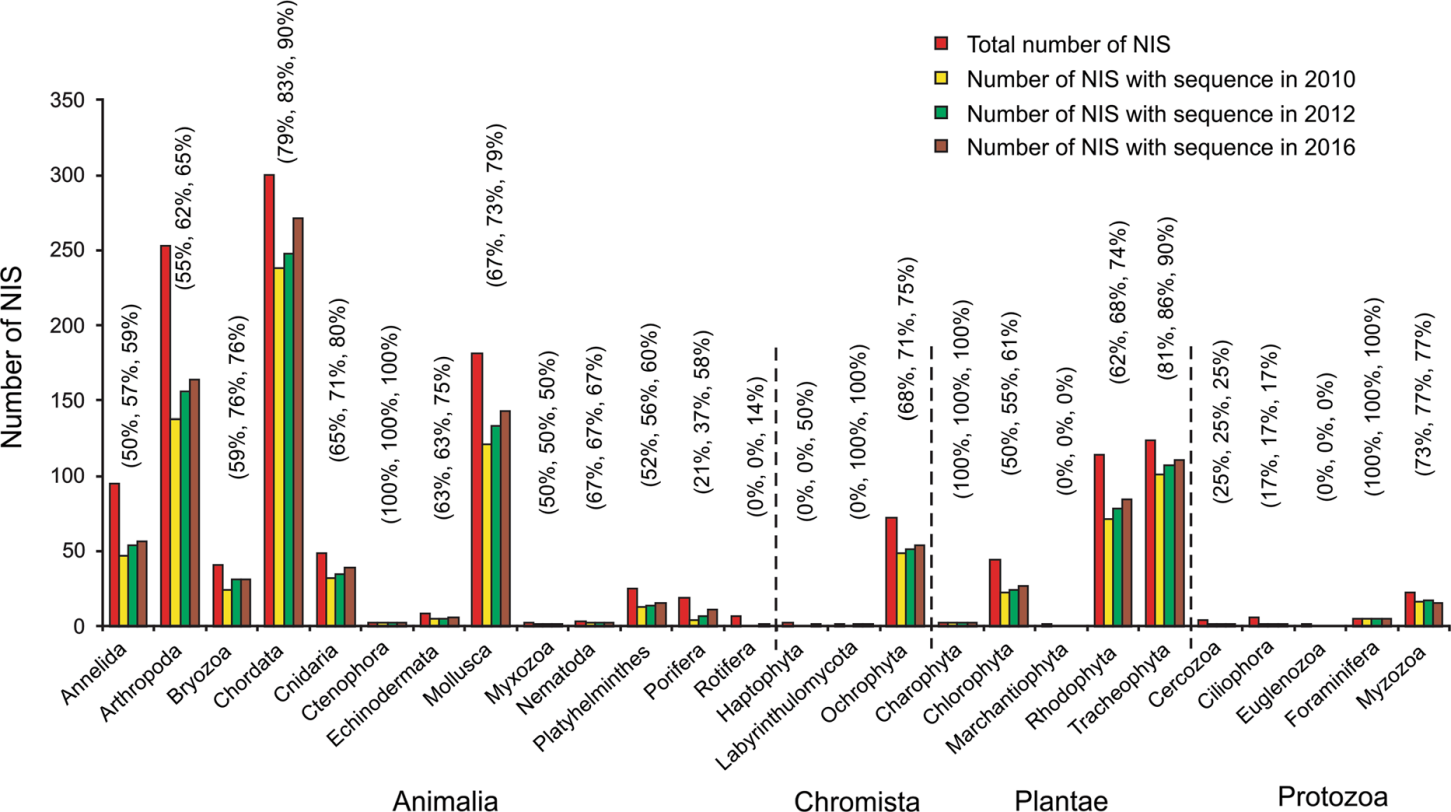
Number of non-indigenous species (NIS) per phylum, and number of NIS with at least one sequence in at least one genetic database in 2010, 2012 and 2016 for aquatic taxa. Percentage cover for 2010, 2012 and 2016 are shown in *brackets*, respectively

Our Thomson’s ISI Web of Knowledge search identified 3101 NIS, of which 1383 (45 %) were aquatic and 1718 (55 %) terrestrial (Fig. 1; Appendix 2 of ESM). Aquatic taxa belonged to four kingdoms: Animalia (71 %), Chromista (5 %), Plantae (21 %) and Protozoa (3 %), consisting of 26 phyla (Figs. 1, 2; Appendix 2 of ESM). The most prevalent aquatic phyla were Annelida (10 %), Arthropoda (26 %), Chordata (30 %) and Mollusca (18 %) in kingdom Animalia, Ochrophyta (96 %) in kingdom Chromista, and Chlorophyta (15 %), Rodophyta (40 %) and Tracheophyta (44 %) in kingdom Plantae. Protozoa was represented by the lowest number of species. When the most dominant Animalia phyla were explored deeper, Malacostraca and Maxillopoda were revealed as the richest Arthropoda classes, Actinopterygii as richest Chordata class, and Bivalvia and Gastopoda as richest Mollusca classes (Appendices 2 and 3 of ESM). In the case of aquatic Plantae, Ulvophyceae and Florideophyceae were dominant classes within Chlorophyta and Rodophyta kingdoms, respectively (Appendices 2 and 3 of ESM).

**Fig. 3:**
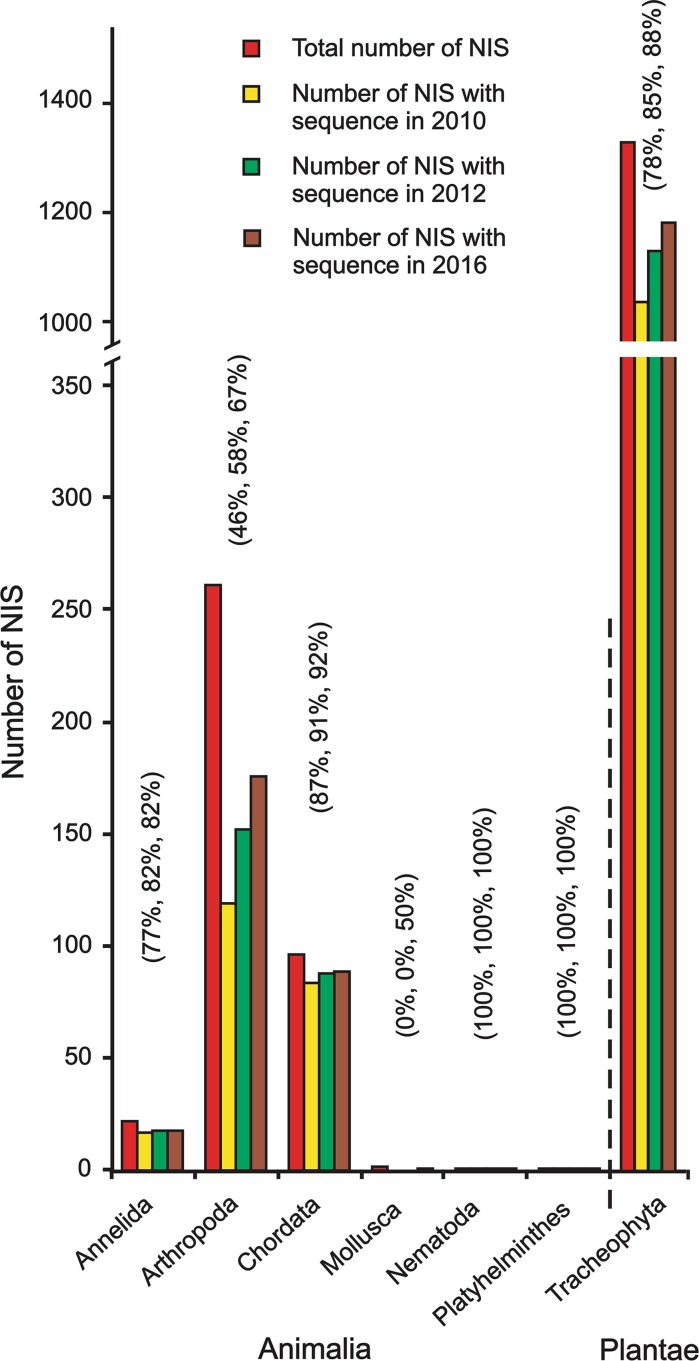
Number of non-indigenous species (NIS) per phylum, and number of NIS with at least one sequence in at least one genetic database in 2010, 2012 and 2016 for terrestrial taxa. Percentage cover for 2010, 2012 and 2016 are shown in *brackets*, respectively

Terrestrial taxa belonged to two kingdoms: Animalia (22 %; having six phyla) and Plantae (78 %; one phylum) (Figs. 1, 3; Appendix 2 of ESM). Arthropoda (68 %) and Chordata (25 %) were the most prevalent Animalia phyla; however, Tracheophyta in Plantae phylum, represented by 1333 species (100 % of terrestrial Plantae), was the most prevalent phylum in both aquatic and terrestrial habitats (Figs. 2, 3; Appendix 2 of ESM). Deeper analyses of terrestrial phyla revealed Insecta as the richest Arthropoda class, and Aves and Mammalia as richest Chordata classes. Liliopsida and Magnoliopsida were the richest Tracheophyta classes (Appendices 2 and 4 of ESM).

### Sequence availability in 2010

**Fig. 4:**
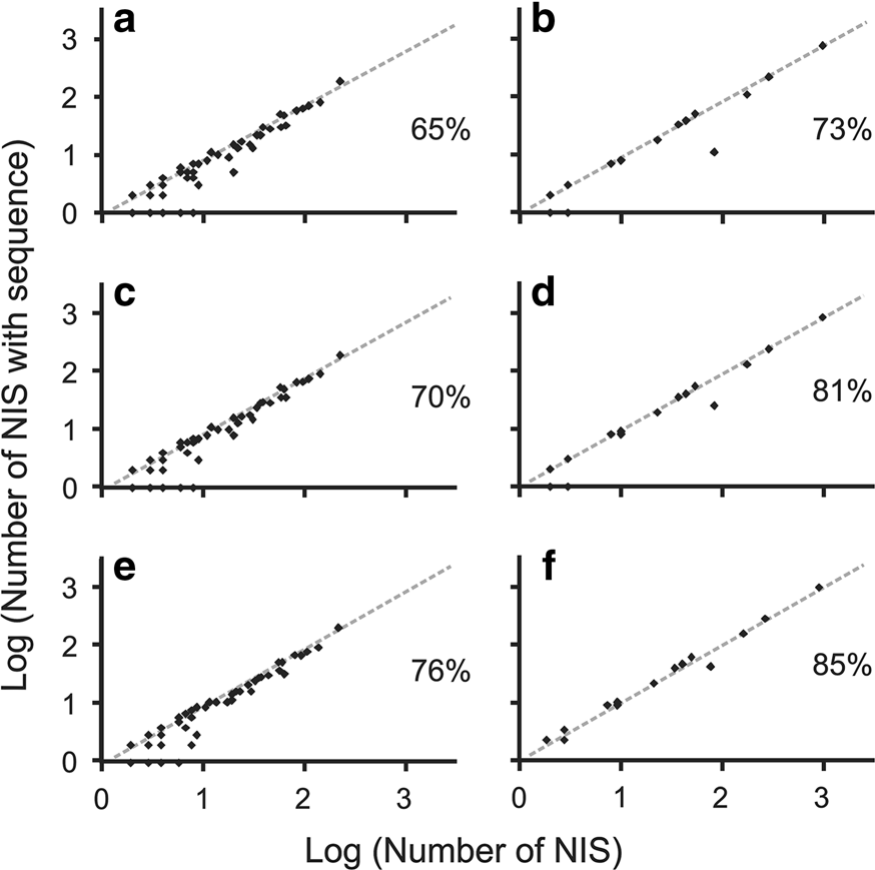
Scatter plots with number of NIS per class on *x*-*axis* and number of NIS with at least one sequence in at least one genetic database per class on *y*-*axis* for aquatic taxa in 2010 (**a**), terrestrial taxa in 2010 (**b**), aquatic taxa in 2012 (**c**), terrestrial taxa in 2012 (**d**), aquatic taxa in 2016 (**e**), and terrestrial taxa in 2016 (**f**). The lines of unity were based on the average percentage of NIS with at least one sequence in at least one genetic database for aquatic taxa in 2010 (**a**), terrestrial taxa in 2010 (**b**), aquatic taxa in 2012 (**c**), terrestrial taxa in 2012 (**d**), aquatic taxa in 2016 (**e**), and terrestrial taxa in 2016 (**f**). Values are log transformed to standardize the data. The average percentages are given for each panel

Eight hundred ninety-five out of 1383 aquatic NIS (65 %) were characterized by at least one sequence (COI, 16S, 18S, ITS, rbcL or matK) in at least one genetic database. All four aquatic kingdoms were similarly represented in the genetic databases; 64, 65, 69, and 61 % of NIS of Animalia, Chromista, Plantae and Protozoa, respectively (Fig. 1; Appendix 2 of ESM). Of 13 Animalia phyla, coverage for ten phyla ranged from 50 to 79 % of NIS; Ctenophora was 100 % covered, while Porifera and Rotifera were 21 and 0 % covered, respectively (Fig. 2; Appendix 2 of ESM). In Chromista phylum, only Ochrophyta had sequences in the genetic databases (68 %), while coverage for Plantae and Protozoa phyla were mixed, ranging from 0 to 100 % (Fig. 2; Appendix 2 of ESM). The majority of aquatic classes were around the average (i.e. 65 %), though twelve classes were not covered at all (Holothuroidea, Turbellaria, Monogononta, Prymnesiophyceae, Labyrinthulomycetes, Xanthophyceae, Marchantiopsida, Compsopogonophyceae, Gromiidea, Ciliatea, Oligohymenophorea, Kinetoplastea; Fig. 4; Appendix 2 of ESM). Classes of the most species-abundant aquatic Animalia and Plantae phyla (i.e. Arthropoda, Chordata, Mollusca, Chlorophyta, Rodophyta, and Tracheophyta) revealed relatively equal sequence representation; most of the classes’ coverage ranged between 50 and 100 % (Appendices 2 and 3 of ESM).

In 2010, out of 1718 terrestrial NIS, 1256 (73 %) were covered by at least one sequence in at least one genetic database (58 % of Animalia and 78 % of Plantae; Fig. 1; Appendix 2 of ESM). Animalia phyla’s coverage ranged from 46 to 100 %, though Mollusca had no sequences in the databases (Fig. 3; Appendix 2 of ESM). Tracheophyta, the only Plantae phylum, was covered for 78 % of species (Fig. 3; Appendix 2 of ESM). The majority of terrestrial classes were around the average (i.e. 73 %), though two classes (Chilopoda and Gastropoda) were not covered at all, and Arachnida was very poorly represented (Fig. 4; Appendix 2 of ESM). Coverage for classes of the most species-abundant terrestrial Animalia and Plantae phyla (i.e. Arthropoda, Chordata, and Tracheophyta) were similar to those for aquatic phyla, with most class coverages ranging between 60 and 100 % (Appendices 2 and 4 of ESM).

### Sequence availability in 2012

Two years later, 71 % of aquatic NIS were represented in the databases; the number of sequences increased to 70, 69, 74 and 63 % for Animalia, Chromista, Plantae, and Protozoa, respectively (Fig. 1; Appendix 2 of ESM). Out of 13 Animalia phyla, new sequences were available for eight phyla (i.e. Annelida, Arthropoda, Bryozoa, Chordata, Cnidaria, Mollusca, Platyhelminthes, and Porifera; Fig. 2; Appendix 2 of ESM). Sequences for two Chromista, three Plantae and one Protozoa phyla also increased (Fig. 2; Appendix 2 of ESM). Representation of most classes was around the average (i.e. 70 %); eleven classes were still not covered at all (Holothuroidea, Turbellaria, Monogononta, Prymnesiophyceae, Xanthophyceae, Marchantiopsida, Compsopogonophyceae, Gromiidea, Ciliatea, Oligohymenophorea, and Kinetoplastea; Fig. 4; Appendix 2 of ESM). Sequence coverage of terrestrial taxa was 81 % in 2012. The number of sequences increased to 68 and 85 % for Animalia and Plantae, respectively (Fig. 1; Appendix 2 of ESM). Out of five Animalia phyla, new sequences were added for three phyla (i.e. Annelida, Arthropoda, and Chordata; Fig. 3; Appendix 2 of ESM). Coverage of Tracheophyta increased to 85 % (Fig. 3; Appendix 2 of ESM). Coverage for the majority of classes was again around the average (i.e. 81 %). Two classes were still not covered (Chilopoda and Gastropoda), as well as Arachnida being less covered than the average (Fig. 4; Appendix 2 of ESM).

### Sequence availability in 2016

In January 2016, 1047 aquatic NIS (76 %) were represented in the databases; the number of species with at least one sequence increased to 743 (75 %), 56 (75 %) and 224 (74 %) for Animalia, Chromista and Plantae, respectively (Fig. 1; Appendix 2 of ESM). No new Protozoa species were covered after 2012 (Fig. 1; Appendix 2 of ESM). New sequences were available for nine Animalia phyla (i.e. Annelida, Arthropoda, Chordata, Cnidaria, Echinodermata, Mollusca, Platyhelminthes, Porifera, and Rotifera; Fig. 2; Appendix 2 of ESM). Sequences for two Chromista and three Plantae phyla also increased (Fig. 2; Appendix 2 of ESM). Representation of most classes was around the average (i.e. 76 %); eight classes were still not covered at all (Turbellaria, Xanthophyceae, Marchantiopsida, Compsopogonophyceae, Gromiidea, Ciliatea, Oligohymenophorea, and Kinetoplastea; Fig. 4; Appendix 2 of ESM). Sequence coverage of terrestrial taxa was 85 % (Fig. 1; Appendix 2 of ESM). The number of sequences increased to 74 and 88 % for Animalia and Plantae, respectively (Fig. 1; Appendix 2 of ESM). Out of five Animalia phyla, new sequences were added for three phyla (i.e. Arthropoda, Chordata, and Mollusca; Fig. 3; Appendix 2 of ESM). Coverage of Tracheophyta increased to 88 % (Fig. 3; Appendix 2 of ESM). Coverage for the majority of classes was again around the average (i.e. 85 %; Fig. 4; Appendix 2 of ESM).

**Fig. 5:**
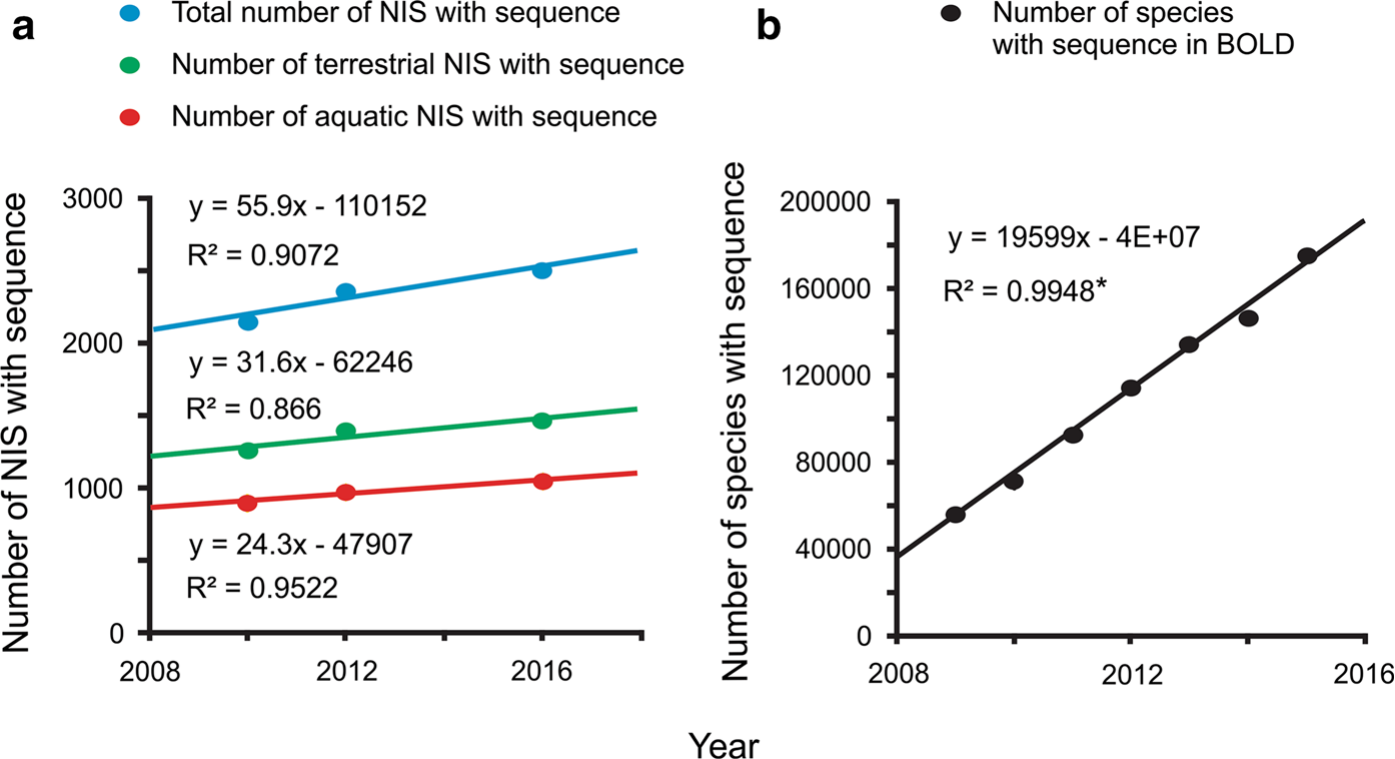
Scatterplot and fitted regression lines with total number of species with at least one sequence in at least one genetic database as the dependent variables and time as the independent variable for all, terrestrial, and aquatic taxa in our study (**a**), and scatterplot and fitted regression line with all species in Barcode of Life Database (BOLD) with at least one sequence as the dependent variable and time as the independent variable (BOLD 2016) (**b**). An *asterisk* denotes significant difference (*P* < 0.05)

Regression analyses revealed no significant increase for either total number of species covered by at least one sequence in at least one database from our NIS list, or for aquatic or terrestrial taxa from our list through time (*P* > 0.05; Fig. 5a). The increase of species with at least one sequence in BOLD independently of indigenous/non-indigenous status was highly significant (*P* < 0.05; Fig. 5b). On average 56 new NIS from our list were covered by at least one sequence per year, while on average sequences for 19,599 new species are entered in BOLD each year (Fig. 5).

### Sequence availability for two or more genes per species

When availability of sequences for two or three genes per species were checked, the species coverage for aquatic taxa dropped from 65 % species covered by at least one sequence in at least one database to 49 % species covered by sequences of at least two genes and to 32 % species covered by sequences of at least three genes, in 2010 (Table 1). The coverage of terrestrial taxa dropped from 78 to 56 (two genes) and 33 % (three genes) in 2010 (Table 1). As more sequences were added to the genetic databases through time, the difference between at least one sequence per species and at least two or three sequences per species declined. The species coverage in 2012 dropped from 71 to 56 (two genes) and 41 % (three genes) for aquatic taxa, and from 85 to 75 (two genes) and 61 % (three genes) for terrestrial taxa, respectively (Table 1). The drop in 2016 was from 76 to 66 and 54 % for aquatic taxa, and from 88 to 85 and 79 % for terrestrial taxa for two and three genes per species, respectively (Table 1).

**Table 1:** Number (#) of species with at least one sequence, at least two sequences, and at least three sequences, in at least one genetic database in 2010, 2012 and 2016 for aquatic and terrestrial taxa

	2010						2012						2016					
	At least one sequence		At least two sequences		At least three sequences		At least one sequence		At least two sequences		At least three sequences		At least one sequence		At least two sequences		At least three sequences	
	#	%	#	%	#	%	#	%	#	%	#	%	#	%	#	%	#	%
Aquatic taxa	895	**65**	672	**49**	430	**32**	975	**71**	780	**56**	567	**41**	1047	**76**	916	**66**	748	**54**
Animalia	627	**64**	483	**49**	316	**32**	688	**70**	550	**56**	400	**41**	743	**75**	650	**66**	524	**53**
Chromista	49	**65**	33	**44**	24	**32**	52	**69**	39	**52**	27	**36**	56	**75**	50	**67**	35	**47**
Plantae	196	**69**	141	**50**	79	**28**	211	**74**	174	**61**	129	**45**	224	**79**	199	**70**	174	**61**
Protozoa	23	**61**	15	**40**	11	**29**	24	**63**	17	**45**	11	**29**	24	**63**	17	**45**	15	**40**
Terrestrial taxa	1256	**73**	914	**52**	530	**31**	1391	**81**	1190	**69**	927	**54**	1460	**85**	1362	**79**	1215	**71**
Animalia	223	**58**	164	**43**	97	**25**	261	**68**	194	**50**	120	**31**	286	**74**	233	**60**	164	**43**
Plantae	1033	**78**	750	**56**	433	**33**	1130	**85**	996	**75**	807	**61**	1174	**88**	1129	**85**	1051	**79**
Total	2151	**69**	1586	**51**	960	**31**	2366	**76**	1970	**64**	1494	**48**	2507	**81**	2278	**74**	1963	**63**

Percentage (%) cover for 2010, 2012 and 2016 are shown in bold

## Discussion

### Availability of sequences for DNA barcoding

As two-thirds of NIS studied in Web of Science are plants and insects (Pyšek et al. 2008), many ecological hypotheses and theories were tested on plants (Blossey and Nötzold 1995; Davis et al. 2000; Minchinton 2002; Keane and Crawley 2002; Mitchell and Power 2003; Richardson and Pyšek 2006). As it is also easier to manipulate experimental design and to conduct experiments and monitoring programs for terrestrial than for aquatic taxa, one might expect that terrestrial taxa would be more extensively studied and consequently better represented by DNA sequences than aquatic taxa. Our study demonstrated, however, that there is little difference between the two. Approximately 75 % of species in almost each aquatic kingdom had at least one sequence in at least one genetic database. Only the coverage of aquatic Protozoa was lower (63 %). Similar coverage was available for terrestrial Animalia while terrestrial Plantae were better covered (88 %). Interestingly, our findings were contrary to the findings of Pyšek et al. (2008) who stated that plant NIS are slightly understudied in the general ecological literature compared to other taxa when number of NIS per taxonomic group has been compared to number of studies per taxonomic group. The same authors found that insects, birds, and reptiles are mildly understudied while crustaceans, molluscs, algae, and mammals are more intensively studied (Pyšek et al. 2008). Our examination of sequence availability is mainly in agreement with Pyšek et al. (2008), though there are some discrepancies. We determined that insect sequence availability was slightly lower than average in both aquatic and terrestrial habitats (59 and 78 %, respectively), while birds and reptiles were better covered (78–100 %). The discrepancy between Pyšek et al. (2008) and our sequence availability results demonstrates that intensity of ecological invasion studies is not clearly correlated to intensity of molecular studies of the same taxa. Encouragingly, some taxonomic groups are mildly understudied in invasion ecology but are well represented in molecular studies with many gene sequences. The opposite pattern has also been observed, however, with more markedly understudied aquatic than terrestrial taxa, particularly those belonging to Chromista and Protozoa kingdoms.

### Deposition of sequences to genetic databases

Between 2010 and 2016, species coverage by DNA sequences increased from 65 and 73 % to 76 and 85 % for aquatic and terrestrial taxa, respectively. Assuming that deposition of sequences to the databases follows a linear function, we expect a reasonably brief period (until 2024) before the majority of terrestrial NIS on our list are sequenced, and a slightly more protracted timeframe (until 2030) before the majority of aquatic NIS are likewise surveyed. We cannot confidently demonstrate that the trend is linear since we have only three time points. The regression analyses determined no significant increase in the number of NIS covered, though deposition of sequences to BOLD irrespective of indigenous/non-indigenous status follows a significant linear trend. As more than three-quarters of NIS on our list are already covered, an optimistic explanation for the lack of a significant increase in NIS coverage may be that the function is saturating and starting to level out. If this is the case, the increase might be significant and much steeper in the period before 2010 than in the last 6 years. However, our list of NIS is not exhaustive, particularly due to uncertainties associated with the status of cryptogenic species, as well as continuous discoveries of new NIS. Bearing in mind that we used the list of NIS assembled in 2010, and did not update it in the consequent years when genetic databases were checked (i.e., 2012 and 2016), it is possible that the rate of increase in NIS coverage is closer to that of total species (irrespective of indigenous/non-indigenous status) in the BOLD than shown by our saturation rates. Furthermore, taking into account the rapid development of molecular techniques and technology, in the near future one may expect the deposition of sequences to follow an exponential rather than linear function. In particular, this might be true for NIS taxa, as studies on invasive species have been rapidly increasing since 1990 (Ricciardi and MacIsaac 2008). In addition, the number of studies of NIS with economic value, such as fishes (e.g. *Cyprinus carpio*, *Salmo trutta*, and *Oncorhynchus mykiss*) and mammals (e.g. *Sus scrofa*), and NIS having severe impact on environment and economy [e.g. *Rattus rattus*, *Dreissena polymorpha*, and *Eichhornia crassipes*; see also Briski et al. (2011) and Trebitz et al. (2015)] is exceptionally high compared to studies of other NIS (MacIsaac et al. 2011). In this study, taxa such as aquatic Malacostraca (many species with environmental or economic impact), Maxillopoda, Bivalvia, and Ulvolaceae (many species of economic value and/or causing impact) and terrestrial Insecta (many species causing environmental or economic impact) demonstrate an exceptionally high trend of sequence deposition. Consequently, while there does not appear to be a strong difference in sequence enrichment between aquatic and terrestrial taxa, we may expect that NIS belonging to particular taxonomic groups would be more rapidly described by gene sequences suitable for DNA barcoding than other species.

### Perspectives on DNA barcoding for detecting NIS

On average 81 % of NIS were covered by sequences in genetic databases, with terrestrial, and in particular plant taxa, having the best coverage. Most taxonomic classes are covered relatively well, though there are still some taxa not covered at all. Our list of NIS is not exhaustive, and many species which are not reported as NIS today may become NIS in the future. So, as long as most of the world biodiversity is not sequenced, we may expect introductions of species that cannot be identified by DNA barcoding. Furthermore, nuclear pseudogenes, heteroplasmy, hybrid introgression, and mitochondrial and plasmid inheritance modes may also reduce the efficiency of DNA barcoding (Hebert et al. 2004; Buhay 2009; Galtier et al. 2009; Hollingsworth et al. 2011; Comtet et al. 2015). Still, the prospect of DNA barcodes for detection and identification of NIS is more promising than traditional morphological identifications. Beside numerous problems connected to morphological identification, taxonomic experts capable to conduct morphological identification are becoming rare, with some taxonomic groups not covered by experts at all (Segers 2008; Ojaveer et al. 2014).

Metabarcoding, which provides millions of sequences from bulk samples, and its application as an environmental DNA (eDNA) monitoring technique that obtains genetic material directly from environmental samples (e.g. water, sediment, and soil) without any obvious signs of biological source material, provides new approaches to population and biodiversity monitoring (Ficetola et al. 2008; Comtet et al. 2015; Goldberg et al. 2015; Thomsen and Willerslev 2015), and invasion ecologists are already developing and adjusting these techniques for early detection of notorious NIS (Turner et al. 2014; Wilson et al. 2014). Use of metabarcoding and multiple markers are expected to increase identification rates, although at least initially, those techniques would increase work- and cost-loads, particularly since there are still developmental technical problems (Zhan et al. 2014a, b; Comtet et al. 2015). Continued enrichment of genetic databases will be required for the effective use of these techniques, including concerted efforts to sequence genes for under-represented groups, irrespective of their economic value or environmental and/or economic impact. In this process, correct species determination (by traditional taxonomy) and proper management of sequence deposition and voucher storage is vital to preserve connections between morphological and molecular data.

## Electronic supplementary material

Below is the link to the electronic supplementary material.

Supplementary material 1 (DOC 4556 kb)
