# Supplementary material for: Are genetic databases sufficiently populated to detect non-indigenous species?
Source: Biol Invasions. 2016 Apr 5;18(7):1911–22. doi: 10.1007/s10530-016-1134-1 (PMC7175672; doi:10.1007/s10530-016-1134-1)

**Electronic Supplementary Material**

Are genetic databases sufficiently populated to detect non-indigenous species?

Elizabeta Briski^1,*^, Sara Ghabooli^2^, Sarah A. Bailey^3^, Hugh J. MacIsaac^2^

^1^GEOMAR, Helmholtz-Zentrum für Ozeanforschung Kiel, D-24105 Kiel, Germany

# ^2^Great Lakes Institute for Environmental Research, University of Windsor, Windsor, Ontario, N9B 3P4, Canada

^3^Great Lakes Laboratory for Fisheries and Aquatic Sciences, Fisheries and Oceans Canada, Burlington, Ontario, L7S 1A1, Canada

# ^*^Corresponding author: E-mails: [**ebriski@geomar.de**](mailto:ebriski@geomar.de), [**elzabriski@yahoo.com**](mailto:elzabriski@yahoo.com); Phone: +49-431-600-1589; Fax: +49-431-600-4402

**Appendix 1** References from Thomson’s ISI (Institute for Science Information) Web of Knowledge 4.0 used to assemble a global list of aquatic and terrestrial non-indigenous species (NIS).

Andreu J, Vila M (2010) Risk analysis of potential invasive plants in Spain. J Nat Conserv 18: 34-44.

Araújo R, Bárbara I, Tibaldo M, Berecibar E, Tapia PD,Pereira R, Santos R, Pinto IS (2009) Checklist of benthic marine algae and cyanobacteria of northern Portugal. Bot Mar 52: 24-46.

Baltz DM (1991) Introduced Fishes in Marine Systems and Inland Seas. Biol Conserv, 56: 151-177.

Barnett DT, Stohlgren TJ, Jarnevich CS, Chong GW, Ericson JA, Davern TR, Simonson SE (2007) The Art and Science of Weed Mapping. Environ Monit Assess 132: 235–252.

## Bradshaw CJA, Giam X, Tan HTW, Brook BW, Sodh NS (2008) ***Threat or invasive status in legumes is related to opposite extremes of the same ecological and life-history attributes.*** J Ecol, 96: 869–883.

Brown GG, James SW (2006) Earthworm biodiversity in São Paulo state, Brazil. Eur J Soil Biol, 42: S145–S149.

Buddenhagen CE, Chimera C, Clifford P (2009) Assessing Biofuel Crop Invasiveness: A Case Study. PLoS ONE 4: e5261.

Castilla JC, Uribe M, Bahamonde N, Clarke M, Desqueyroux-Faúndez R, Kong I, Moyano H, Rozbaczylo N, Santelices B, Valdovinos C, Zavala P (2005) Down under the southeastern Pacific: marine non-indigenous species in Chile. Biol Invasions 7: 213–232.

Dash ST, Hooper-Bùi LM (2008) Species Diversity of Ants (Hymenoptera: Formicidae) in Louisiana. Ann Entomol Soc Am 101: 1056-1066.

de Almeida CFBR, Ramos MA, de Amorim ELC, de Albuquerque US (2010) A comparison of knowledge about medicinal plants for three rural communities in the semi-arid region of northeast of Brazil. J Ethnopharmacol 127: 674-684.

Devin S, Bollache L, Noël P-Y, Beisel J-N (2005) Patterns of biological invasions in French freshwater systems by non-indigenous macroinvertebrates. Hydrobiologia 551: 137–146.

deWaard JR, Landry J-F, Christian Schmidt B, Derhousoff J, McLean JA, Humble LM (2009) In the dark in a large urban park: DNA barcodes illuminate cryptic and introduced moth species. Biodivers Conserv 18: 3825–3839.

Eyles AC (1999) Introduced Mirinae of New Zealand (Hemiptera: Miridae). New Zeal J Zool 26: 355-372.

Frank JH, McCoy ED (2007) The risk of classical biological control in Florida. Biol Control 41: 151–174.

Gherardi F, Bertolino S, Bodon M, Casellato S, Cianfanelli S, Ferraguti M, Lori E, Mura G, Nocita A, Riccardi N, Rossetti G, Rota E, Scalera R, Zerunian S, Tricarico E (2008) Animal xenodiversity in Italian inland waters: distribution, modes of arrival, and pathway. Biol Invasions 10: 435-454.

Hayes KR, Sliwa C (2003) Identifying potential marine pests -a deductive approach applied to Australia. Mar Pollut Bull 46: 91–98.

Hewitt CL, Campbell ML, Thresher RE, Martin RB, Boyd S, Cohen BF, Currie DR, Gomon MF, Keough MJ, Lewis JA, Lockett MM, Mays N, McArthur MA, O’Hara TD, Poore GCB, Ross DJ, Storey MJ, Watson JE, Wilson RS (2004) Introduced and cryptogenic species in Port Phillip Bay, Victoria, Australia. Mar Biol 144: 183–202.

Johnson JA, Ward PS (2002) Biogeography and endemism of ants

(Hymenoptera: Formicidae) in Baja California, Mexico: a first overview. J Biogeogr 29: 1009–1026.

Karabin JE (2011) Does invasion of Vallisneria spiralis L. promote appearance of rare and new rotifer (Rotifera) species in littoral of the lakes heated by power station (Konin Lakes, W. Poland)? Pol J Ecol 59: 201-207.

Keller RP, zu Ermgassen PSE, Aldridge DC. (2009) Vectors and timing of freshwater invasions in Great Britain. Conserv Biol 23: 1526-1534.

Křivánek M, Pyšek P (2006) Predicting invasions by woody species in a temperate zone: a test of three risk assessment schemes in the Czech Republic (Central Europe). Divers Distrib 12: 319–327.

# Leppäkoski E, Gollasch S, Gruszka P, Ojaveer H, Olenin S, Panov V (2002) The Baltic—a sea of invaders. **Can J Fish Aquat Sci** 59: 1175-1188.

Leung GPC, Hau BCH, Corlett RT (2009) Exotic plant invasion in the highly degraded upland landscape of Hong Kong, China. Biodivers Conserv 18: 191–202.

Majka CG, Anderson RS, McCorquodale DB (2007a) The weevils (Coleoptera: Curculionoidea) of the Maritime Provinces of Canada, II: New records form Nova Scotia and Prince Edward Island and regional zoogeography. Can Entomol 139: 397-442.

Majka CG, Anderson RS, McAlpine DF, Webster RP (2007b) The weevils (Coleoptera: Curculionoidea) of the Maritime Provinces of Canada, II: New records form New Brunswick. Can Entomol 139: 378-396.

Mastrototaro F, D’Onghia G, Tursi A (2008) Spatial and seasonal distribution of ascidians in a semi-enclosed basin of the Mediterranean Sea. J Mar Biol Assoc UK 88: 1053-1061.

Morrison LW (1998) A Review of Bahamian Ant (Hymenoptera: Formicidae) Biogeography. J Biogeogr 25: 561-571.

Molnar JL, Gamboa RL, Revenga C, Spalding MD (2008) Assessing the global threat of invasive species to marine biodiversity. Front Ecol Environ 6: 485-492.

Nafría JMN, Durante MPM, Ortego J, Fernández MVS (2007) The genus Uroleucon (Hemiptera: Aphididae: Macrosiphini) in Argentina, with descriptions of five species. Can Entomol 139: 154-178.

Navia D, Ochoa R, Welbourn C, Ferragut F (2010) Adventive eriophyoid mites: a global review of their impact, pathways, prevention and challenges. Exp Appl Acarol 51: 225-255.

# Ou J, Lu C, O'Toole DK (2008) A risk assessment system for alien plant bio-invasion in Xiamen, China. J Environ Sci 20: 989–997.

Paini DR, Worner SP, Cook DC, De Barro PJ, Thomas MB (2010) Using a self-organizing map to predict invasive species: sensitivity to data errors and a comparison with expert opinion. J Appl Ecol 47: 290-298.

Piasecki W, Kuźmińska E (2007) Developmental stages of Achthers percarum (Crustacea: Copepoda), parasitic on European perch, Perca fluviatilis (Actinopterygii: Perciformes). Acta Ichthyol Piscat 37: 117–128.

Protopopova VV, Shevera MV, Mosyakin SL (2006) Deliberate and unintentional introduction of invasive weeds: A case study of the alien flora of Ukraine. Euphytica, 148: 17–33.

Puillandre N, Dupas S, Dangles O, Zeddam J-L, Capdevielle-Dulac C, Barbin K, Torres-Leguizamon M, Silvain J-F (2008) Genetic bottleneck in invasive species: the potato tuber moth adds to the list. Biol Invasions 10: 319–333.

Pyšek P (2005) Survival rates in the Czech Republic of introduced plants known as wool aliens. Biol Invasions 7: 567–576.

Ray GL (2005a) Invasive Marine and Estuarine Animals of Hawai’i and other Pacific Islands. Aquatic Nuisance Species Research Program, ERDC/TN ANSRP-05-3.

Ray GL (2005b) Invasive Marine and Estuarine Animals of Hawai’i and other Pacific Islands. Aquatic Nuisance Species Research Program, ERDC/TN ANSRP-05-1.

Ricciardi A (2006) Patterns of invasion in the Laurentian Great Lakes in relation to changes in vector activity. Divers Distrib 12: 425-433.

Rodrigo Rojas JM, Rodríguez OS (2008) Diversidad y abundancia ictiofaunística del río Grande de Térraba, sur de Costa Rica. Rev Biol Trop 56: 1429-1447.

Ruiz GM, Fofonoff PW, Carlton JT, Wonham MJ, Hines AH (2000) Invasion of coastal marine communities in North America: apparent patterns, processes, and biases. Ann Rev Ecol Syst 31: 481-531.

Sfriso A, Curiel D (2007) Check-list of seaweeds recorded in the last 20 years in Venice lagoon, and a comparison with the previous records. Bot Mar 50: 22–58.

Shafland PL, Gestring KB, Stanford MS (2008) Categorizing introduced fishes collected from public waters. Southeast Nat 7: 627–636.

Smallwood KS (1992) A rating system for potential exotic bird and mammal pests. Biol Conserv 62: 149-159.

Strayer DL (2010) Alien species in fresh waters: ecological effects, interactions with other stressors, and prospects for the future. Freshwater Biol 55: 152–174.

Streftaris N, Zentos A (2006) Alien marine species in the Mediterranean - the 100 ‘worst invasives’ and their Impact. Mediterr Mar Sci 7: 87-118.

Tsiamis K, Panayotidis P, Zenetos A (2008) Alien marine macrophytes in Greece: a review. Bot Mar 51: 237–246.

Van Wilgen BW, Nel JL, Rouget M (2007) Invasive alien plants and South African rivers: a proposed approach to the prioritization of control operations. Freshwater Biol 52: 711–723.

Verreycken H, Anseeuw D, Van Thuyne G, Quataert P, Belpaire C (2007) The non-indigenous freshwater fishes of Flanders (Belgium): review, status and trends over the last decade. J Fish Biol 71: 160–172.

Villaseñor JL, Espinosa-Garcia FJ (2004) The alien flowering plants of Mexico. Divers Distrib 10: 113–123.

Weber E, Gut D (2005) A survey of weeds that are increasingly spreading in Europe. Agron Sustain Dev 25: 109–121.

# [Wonham](http://link.springer.com/search?facet-author="Marjorie+J.+Wonham") MJ, Carlton JT (2005) Trends in marine biological invasions at local and regional scales: the Northeast Pacific Ocean as a model system. Biol Invasions 7: 369-392.

Wu S-H, Hsieh C-F, Chaw S-M, Rejmánek M (2004) Plant invasions in Taiwan: Insights from the flora of casual and naturalized alien species. Divers Distrib 10: 349–362.

Zenetos A, Çinar ME, Pancucci-Papadopoulou MA, Harmelin JG, Furnari G, Andaloro F, Bellou N, Streftaris N, Zibrowius H (2005) Annotated list of marine alien species in the Mediterranean with records of the worst invasive species. Mediterr Mar Sci 6: 63-118.

Zomlefer WB, Giannasi DE, Echols LS (2010) Vascular Plant Flora of Kennesaw Mountain National Battlefi eld Park, Cobb County, Georgia. Southeast Nat 9: 129-164.

**APPENDIX 2** Number of non-indigenous species (NIS) per kingdom, phylum and class recovered by Thomson’s ISI (Institute for Science Information) Web of Knowledge 4.0 and Global Invasive Species Database of the Invasive Species Specialist Group (ISSG, 2010) search. Number of sequences for cytochrome *c* oxidase subunit I (COI), small subunit ribosomal16S rDNA (16S), small subunit ribosomal 18S rDNA (18S), internal transcribed spacer (ITS), ribulose-bisphosphate carboxylase (rbcL), and maturase K (matK) genes recovered from GenBank and Barcode of Life Database (BOLD) in 2010, 2012 and 2016 are also shown. Percentage of species (%) covered by at least one sequence in at least one genetic database for 2010, 2012 and 2016 is shown in bold.

|  |  |  | 2010 | | | | | | | | |  | 2012 | | | | | | | | |
| --- | --- | --- | --- | --- | --- | --- | --- | --- | --- | --- | --- | --- | --- | --- | --- | --- | --- | --- | --- | --- | --- |
|  |  | # of NIS | GenBank | | | | | | BOLD | Total # | % |  | GenBank | | | | | | BOLD | Total # | % |
|  |  |  | COI | 16S | 18S | ITS | rbcL | matK | COI |  |  |  | COI | 16S | 18S | ITS | rbcL | matK | COI |  |  |
| Aquatic taxa |  | 1383 | 471 | 393 | 388 | 246 | 187 | 56 | 511 | 895 | **65** |  | 584 | 482 | 520 | 332 | 222 | 92 | 553 | 975 | **71** |
| Animalia | | 985 | 416 | 359 | 281 | 119 | 0 | 0 | 422 | 627 | **64** |  | 504 | 438 | 366 | 172 | 0 | 0 | 439 | 688 | **70** |
| Annelida | | 95 | 23 | 20 | 34 | 5 | 0 | 0 | 18 | 47 | **49** |  | 31 | 26 | 42 | 10 | 0 | 0 | 19 | 54 | **57** |
|  | Clitellata | 28 | 8 | 9 | 7 | 0 | 0 | 0 | 7 | 14 | **50** |  | 13 | 13 | 12 | 4 | 0 | 0 | 7 | 17 | **61** |
|  | Hirudinea | 2 | 2 | 0 | 2 | 0 | 0 | 0 | 2 | 2 | **100** |  | 2 | 0 | 2 | 0 | 0 | 0 | 2 | 2 | **100** |
|  | Polychaeta | 65 | 13 | 11 | 25 | 5 | 0 | 0 | 9 | 31 | **48** |  | 16 | 13 | 28 | 6 | 0 | 0 | 10 | 35 | **54** |
| Arthropoda | | 253 | 97 | 77 | 66 | 31 | 0 | 0 | 93 | 138 | **55** |  | 111 | 92 | 87 | 41 | 0 | 0 | 104 | 156 | **62** |
|  | Branchiopoda | 23 | 9 | 9 | 8 | 2 | 0 | 0 | 11 | 16 | **70** |  | 10 | 11 | 9 | 3 | 0 | 0 | 11 | 16 | **70** |
|  | Insecta | 17 | 5 | 3 | 4 | 3 | 0 | 0 | 4 | 8 | **47** |  | 5 | 5 | 5 | 4 | 0 | 0 | 5 | 9 | **53** |
|  | Malacostraca | 143 | 61 | 51 | 32 | 15 | 0 | 0 | 57 | 80 | **56** |  | 72 | 61 | 45 | 20 | 0 | 0 | 64 | 91 | **64** |
|  | Maxillopoda | 58 | 19 | 11 | 17 | 10 | 0 | 0 | 18 | 29 | **50** |  | 21 | 12 | 23 | 13 | 0 | 0 | 21 | 35 | **60** |
|  | Merostomata | 1 | 1 | 1 | 1 | 1 | 0 | 0 | 1 | 1 | **100** |  | 1 | 1 | 1 | 1 | 0 | 0 | 1 | 1 | **100** |
|  | Ostracoda | 8 | 0 | 0 | 2 | 0 | 0 | 0 | 0 | 2 | **25** |  | 0 | 0 | 2 | 0 | 0 | 0 | 0 | 2 | **25** |
|  | Pycnogonida | 3 | 2 | 2 | 2 | 0 | 0 | 0 | 2 | 2 | **67** |  | 2 | 2 | 2 | 0 | 0 | 0 | 2 | 2 | **67** |
| Bryozoa | | 41 | 16 | 14 | 16 | 0 | 0 | 0 | 16 | 24 | **59** |  | 23 | 21 | 19 | 2 | 0 | 0 | 16 | 31 | **76** |
|  | Gymnolaemata | 36 | 15 | 12 | 14 | 0 | 0 | 0 | 16 | 21 | **58** |  | 22 | 19 | 17 | 2 | 0 | 0 | 16 | 28 | **78** |
|  | Phylactolaemata | 2 | 1 | 2 | 1 | 0 | 0 | 0 | 0 | 2 | **100** |  | 1 | 2 | 1 | 0 | 0 | 0 | 0 | 2 | **100** |
|  | Not defined | 3 | 0 | 0 | 1 | 0 | 0 | 0 | 0 | 1 | **33** |  | 0 | 0 | 1 | 0 | 0 | 0 | 0 | 1 | **33** |
| Chordata | | 300 | 159 | 144 | 66 | 31 | 0 | 0 | 206 | 238 | **79** |  | 207 | 174 | 92 | 52 | 0 | 0 | 207 | 248 | **83** |
|  | Actinopterygii | 225 | 120 | 126 | 41 | 27 | 0 | 0 | 164 | 185 | **82** |  | 162 | 154 | 63 | 43 | 0 | 0 | 164 | 193 | **86** |
|  | Amphibia | 8 | 3 | 5 | 2 | 0 | 0 | 0 | 5 | 6 | **75** |  | 5 | 5 | 2 | 1 | 0 | 0 | 5 | 6 | **75** |
|  | Ascidiacea | 45 | 20 | 3 | 17 | 3 | 0 | 0 | 19 | 27 | **60** |  | 22 | 3 | 20 | 5 | 0 | 0 | 19 | 28 | **62** |
|  | Aves | 11 | 8 | 3 | 2 | 0 | 0 | 0 | 10 | 10 | **91** |  | 9 | 3 | 2 | 0 | 0 | 0 | 10 | 10 | **91** |
|  | Cephalaspidomorphi | 1 | 1 | 1 | 1 | 1 | 0 | 0 | 1 | 1 | **100** |  | 1 | 1 | 1 | 1 | 0 | 0 | 1 | 1 | **100** |
|  | Chondrichthyes | 3 | 3 | 2 | 0 | 0 | 0 | 0 | 3 | 3 | **100** |  | 3 | 2 | 0 | 1 | 0 | 0 | 3 | 3 | **100** |
|  | Mammalia | 5 | 3 | 3 | 2 | 0 | 0 | 0 | 3 | 4 | **80** |  | 4 | 4 | 3 | 1 | 0 | 0 | 4 | 5 | **80** |
|  | Reptilia | 2 | 1 | 1 | 1 | 0 | 0 | 0 | 1 | 2 | **100** |  | 1 | 2 | 1 | 0 | 0 | 0 | 1 | 2 | **100** |
| Cnidaria | | 49 | 13 | 21 | 17 | 6 | 0 | 0 | 10 | 32 | **65** |  | 13 | 26 | 23 | 8 | 0 | 0 | 10 | 35 | **71** |
|  | Anthozoa | 10 | 4 | 3 | 3 | 1 | 0 | 0 | 3 | 7 | **70** |  | 4 | 4 | 4 | 1 | 0 | 0 | 2 | 7 | **70** |
|  | Hydrozoa | 33 | 5 | 17 | 13 | 4 | 0 | 0 | 4 | 21 | **64** |  | 5 | 20 | 15 | 5 | 0 | 0 | 4 | 23 | **70** |
|  | Scyphozoa | 6 | 4 | 1 | 1 | 1 | 0 | 0 | 3 | 4 | **67** |  | 4 | 2 | 4 | 2 | 0 | 0 | 4 | 5 | **83** |
| Ctenophora | | 2 | 1 | 0 | 2 | 2 | 0 | 0 | 0 | 2 | **100** |  | 1 | 0 | 2 | 2 | 0 | 0 | 0 | 2 | **100** |
|  | Nuda | 1 | 1 | 0 | 1 | 1 | 0 | 0 | 0 | 1 | **100** |  | 1 | 0 | 1 | 1 | 0 | 0 | 0 | 1 | **100** |
|  | Tentaculata | 1 | 0 | 0 | 1 | 1 | 0 | 0 | 0 | 1 | **100** |  | 0 | o | 1 | 1 | 0 | 0 | 0 | 1 | **100** |
| Echinodermata | | 8 | 5 | 2 | 2 | 0 | 0 | 0 | 4 | 5 | **63** |  | 5 | 3 | 3 | 1 | 0 | 0 | 5 | 5 | **63** |
|  | Asteroidea | 5 | 4 | 2 | 2 | 0 | 0 | 0 | 3 | 4 | **80** |  | 4 | 3 | 3 | 1 | 0 | 0 | 4 | 4 | **80** |
|  | Holothuroidea | 1 | 0 | 0 | 0 | 0 | 0 | 0 | 0 | 0 | **0** |  | 0 | 0 | 0 | 0 | 0 | 0 | 0 | 0 | **0** |
|  | Ophiuroidea | 2 | 1 | 0 | 0 | 0 | 0 | 0 | 1 | 1 | **50** |  | 1 | 0 | 0 | 0 | 0 | 0 | 1 | 1 | **50** |
| Mollusca | | 181 | 91 | 77 | 63 | 34 | 0 | 0 | 69 | 121 | **67** |  | 101 | 92 | 77 | 43 | 0 | 0 | 72 | 133 | **73** |
|  | Bivalvia | 83 | 38 | 29 | 36 | 25 | 0 | 0 | 28 | 57 | **69** |  | 42 | 37 | 45 | 30 | 0 | 0 | 30 | 65 | **78** |
|  | Cephalopoda | 2 | 2 | 2 | 0 | 0 | 0 | 0 | 2 | 2 | **100** |  | 2 | 2 | 0 | 0 | 0 | 0 | 2 | 2 | **100** |
|  | Gastropoda | 96 | 51 | 46 | 27 | 9 | 0 | 0 | 39 | 62 | **65** |  | 57 | 53 | 32 | 13 | 0 | 0 | 40 | 66 | **69** |
| Myxozoa | | 2 | 0 | 0 | 1 | 1 | 0 | 0 | 0 | 1 | **50** |  | 0 | 0 | 1 | 1 | 0 | 0 | 0 | 1 | **50** |
|  | Myxosporea | 2 | 0 | 0 | 1 | 1 | 0 | 0 | 0 | 1 | **50** |  | 0 | 0 | 1 | 1 | 0 | 0 | 0 | 1 | **50** |
| Nematoda | | 3 | 2 | 1 | 2 | 1 | 0 | 0 | 2 | 2 | **67** |  | 2 | 1 | 2 | 1 | 0 | 0 | 2 | 2 | **67** |
|  | Secernentea | 3 | 2 | 1 | 2 | 1 | 0 | 0 | 2 | 2 | **67** |  | 2 | 1 | 2 | 1 | 0 | 0 | 2 | 2 | **67** |
| Platyhelminthes | | 25 | 5 | 1 | 10 | 8 | 0 | 0 | 3 | 13 | **52** |  | 6 | 1 | 12 | 10 | 0 | 0 | 3 | 14 | **56** |
|  | Cestoidea | 7 | 2 | 0 | 2 | 2 | 0 | 0 | 1 | 4 | **57** |  | 3 | 0 | 4 | 2 | 0 | 0 | 1 | 5 | **71** |
|  | Monogenea | 77 | 1 | 1 | 6 | 5 | 0 | 0 | 1 | 6 | **86** |  | 1 | 1 | 6 | 6 | 0 | 0 | 1 | 6 | **86** |
|  | Trematoda | 6 | 2 | 0 | 2 | 1 | 0 | 0 | 1 | 3 | **50** |  | 2 | 0 | 2 | 2 | 0 | 0 | 1 | 3 | **50** |
|  | Turbellaria | 5 | 0 | 0 | 0 | 0 | 0 | 0 | 0 | 0 | **0** |  | 0 | 0 | 0 | 0 | 0 | 0 | 0 | 0 | **0** |
| Porifera | | 19 | 4 | 2 | 2 | 0 | 0 | 0 | 1 | 4 | **21** |  | 4 | 2 | 6 | 1 | 0 | 0 | 1 | 7 | **37** |
|  | Demospongiae | 19 | 4 | 2 | 2 | 0 | 0 | 0 | 1 | 4 | **21** |  | 4 | 2 | 6 | 1 | 0 | 0 | 1 | 7 | **37** |
| Rotifera | | 7 | 0 | 0 | 0 | 0 | 0 | 0 | 0 | 0 | **0** |  | 0 | 0 | 0 | 0 | 0 | 0 | 0 | 0 | **0** |
|  | Monogononta | 7 | 0 | 0 | 0 | 0 | 0 | 0 | 0 | 0 | **0** |  | 0 | 0 | 0 | 0 | 0 | 0 | 0 | 0 | **0** |
| Chromista | | 75 | 14 | 12 | 17 | 21 | 39 | 8 | 17 | 49 | **65** |  | 19 | 16 | 30 | 26 | 42 | 8 | 17 | 52 | **69** |
| Haptophyta | | 2 | 0 | 0 | 0 | 0 | 0 | 0 | 0 | 0 | **0** |  | 0 | 0 | 0 | 0 | 0 | 0 | 0 | 0 | **0** |
|  | Prymnesiophyceae | 2 | 0 | 0 | 0 | 0 | 0 | 0 | 0 | 0 | **0** |  | 0 | 0 | 0 | 0 | 0 | 0 | 0 | 0 | **0** |
| Labyrinthulomycota | | 1 | 0 | 0 | 0 | 0 | 0 | 0 | 0 | 0 | **0** |  | 0 | 0 | 1 | 1 | 0 | 0 | 0 | 1 | **100** |
|  | Labyrinthulomycetes | 1 | 0 | 0 | 0 | 0 | 0 | 0 | 0 | 0 | **0** |  | 0 | 0 | 1 | 1 | 0 | 0 | 0 | 1 | **100** |
| Ochrophyta | | 72 | 14 | 12 | 17 | 21 | 39 | 8 | 17 | 49 | **68** |  | 19 | 16 | 29 | 25 | 42 | 8 | 17 | 51 | **71** |
|  | Bacillariophyceae | 7 | 0 | 1 | 2 | 3 | 0 | 0 | 2 | 3 | **43** |  | 0 | 2 | 4 | 4 | 0 | 0 | 2 | 5 | **71** |
|  | Chrysophyceae | 1 | 1 | 1 | 1 | 1 | 1 | 1 | 0 | 1 | **100** |  | 1 | 1 | 1 | 1 | 1 | 1 | 0 | 1 | **100** |
|  | Coscinodiscophyceae | 21 | 5 | 5 | 6 | 6 | 10 | 1 | 2 | 12 | **57** |  | 7 | 6 | 11 | 7 | 11 | 1 | 2 | 12 | **57** |
|  | Pelagophyceae | 1 | 1 | 1 | 0 | 0 | 1 | 0 | 0 | 1 | **100** |  | 1 | 1 | 1 | 1 | 1 | 0 | 0 | 1 | **100** |
|  | Phaeophyceae | 38 | 6 | 2 | 6 | 9 | 25 | 5 | 13 | 29 | **76** |  | 8 | 3 | 10 | 10 | 27 | 5 | 13 | 29 | **76** |
|  | Raphidophyceae | 3 | 1 | 2 | 2 | 2 | 2 | 1 | 0 | 3 | **100** |  | 2 | 3 | 2 | 2 | 2 | 1 | 0 | 3 | **100** |
|  | Xanthophyceae | 1 | 0 | 0 | 0 | 0 | 0 | 0 | 0 | 0 | **0** |  | 0 | 0 | 0 | 0 | 0 | 0 | 0 | 0 | **0** |
| Plantae | | 285 | 32 | 17 | 74 | 91 | 137 | 42 | 64 | 196 | **69** |  | 50 | 23 | 106 | 118 | 169 | 88 | 211 | 211 | **74** |
| Charophyta | | 2 | 0 | 2 | 1 | 0 | 2 | 1 | 0 | 2 | **100** |  | 0 | 2 | 2 | 0 | 2 | 2 | 0 | 2 | **100** |
|  | Charophyceae | 2 | 0 | 2 | 1 | 0 | 2 | 1 | 0 | 2 | **100** |  | 0 | 2 | 2 | 0 | 2 | 2 | 0 | 2 | **100** |
| Chlorophyta | | 44 | 3 | 0 | 10 | 8 | 15 | 0 | 4 | 22 | **50** |  | 2 | 2 | 14 | 11 | 18 | 0 | 6 | 24 | **55** |
|  | Bryopsidophyceae | 13 | 1 | 0 | 3 | 2 | 7 | 0 | 1 | 9 | **69** |  | 1 | 2 | 3 | 3 | 8 | 0 | 3 | 9 | **69** |
|  | Trebouxiophyceae | 1 | 0 | 0 | 1 | 0 | 1 | 0 | 0 | 1 | **100** |  | 0 | 0 | 1 | 0 | 1 | 0 | 0 | 11 | **100** |
|  | Ulvophyceae | 30 | 2 | 0 | 6 | 6 | 7 | 0 | 3 | 12 | **40** |  | 1 | 0 | 10 | 8 | 9 | 0 | 3 | 14 | **47** |
| Marchantiophyta | | 1 | 0 | 0 | 0 | 0 | 0 | 0 | 0 | 0 | **0** |  | 0 | 0 | 0 | 0 | 0 | 0 | 0 | 0 | **0** |
|  | Marchantiopsida | 1 | 0 | 0 | 0 | 0 | 0 | 0 | 0 | 0 | **0** |  | 0 | 0 | 0 | 0 | 0 | 0 | 0 | 0 | **0** |
| Rodophyta | | 114 | 16 | 6 | 21 | 16 | 57 | 1 | 34 | 71 | **62** |  | 35 | 8 | 33 | 22 | 61 | 1 | 39 | 78 | **68** |
|  | Bangiophyceae | 1 | 1 | 0 | 1 | 0 | 1 | 0 | 0 | 1 | **100** |  | 1 | 0 | 1 | 1 | 1 | 0 | 0 | 1 | **100** |
|  | Compsopogonophyceae | 1 | 0 | 0 | 0 | 0 | 0 | 0 | 0 | 0 | **0** |  | 0 | 0 | 0 | 0 | 0 | 0 | 0 | 0 | **0** |
|  | Florideophyceae | 110 | 15 | 6 | 19 | 16 | 56 | 1 | 34 | 69 | **63** |  | 34 | 7 | 31 | 21 | 60 | 1 | 38 | 75 | **68** |
|  | Rhodellophyceae | 2 | 0 | 0 | 1 | 0 | 0 | 0 | 0 | 1 | **50** |  | 0 | 1 | 1 | 0 | 0 | 0 | 1 | 2 | **100** |
| Tracheophyta | | 124 | 13 | 9 | 42 | 67 | 63 | 40 | 26 | 101 | **82** |  | 13 | 11 | 57 | 85 | 88 | 75 | 43 | 107 | **86** |
|  | Liliopsida | 57 | 5 | 3 | 16 | 28 | 32 | 21 | 18 | 49 | **86** |  | 5 | 3 | 28 | 39 | 43 | 38 | 23 | 53 | **93** |
|  | Magnoliopsida | 62 | 8 | 4 | 22 | 35 | 26 | 18 | 8 | 47 | **76** |  | 8 | 6 | 25 | 41 | 40 | 36 | 20 | 49 | **79** |
|  | Polypodiopsida | 5 | 0 | 2 | 4 | 4 | 5 | 1 | 0 | 5 | **100** |  | 0 | 2 | 4 | 5 | 5 | 1 | 0 | 5 | **100** |
| Protozoa | | 38 | 9 | 5 | 16 | 15 | 4 | 1 | 8 | 23 | **61** |  | 11 | 5 | 18 | 16 | 4 | 1 | 9 | 24 | **63** |
| Cercozoa | | 4 | 0 | 1 | 0 | 0 | 0 | 0 | 0 | 1 | **25** |  | 0 | 1 | 0 | 0 | 0 | 0 | 0 | 1 | **25** |
|  | Ascetosporea | 1 | 0 | 1 | 0 | 0 | 0 | 0 | 0 | 1 | **100** |  | 0 | 1 | 0 | 0 | 0 | 0 | 0 | 1 | **100** |
|  | Gromiidea | 3 | 0 | 0 | 0 | 0 | 0 | 0 | 0 | 0 | **0** |  | 0 | 0 | 0 | 0 | 0 | 0 | 0 | 0 | **0** |
| Ciliophora | | 6 | 0 | 0 | 1 | 0 | 1 | 1 | 0 | 1 | **17** |  | 0 | 0 | 1 | 0 | 1 | 1 | 0 | 1 | **17** |
|  | Ciliatea | 2 | 0 | 0 | 0 | 0 | 0 | 0 | 0 | 0 | **0** |  | 0 | 0 | 0 | 0 | 0 | 0 | 0 | 0 | **0** |
|  | Kinetofragminophora | 1 | 0 | 0 | 1 | 0 | 1 | 1 | 0 | 1 | **100** |  | 0 | 0 | 1 | 0 | 1 | 1 | 0 | 1 | **100** |
|  | Oligohymenophorea | 3 | 0 | 0 | 0 | 0 | 0 | 0 | 0 | 0 | **0** |  | 0 | 0 | 0 | 0 | 0 | 0 | 0 | 0 | **0** |
| Euglenozoa | | 1 | 0 | 0 | 0 | 0 | 0 | 0 | 0 | 0 | **0** |  | 0 | 0 | 0 | 0 | 0 | 0 | 0 | 0 | **0** |
|  | Kinetoplastea | 1 | 0 | 0 | 0 | 0 | 0 | 0 | 0 | 0 | **0** |  | 0 | 0 | 0 | 0 | 0 | 0 | 0 | 0 | **0** |
| Foraminifera | | 5 | 0 | 1 | 4 | 1 | 1 | 0 | 0 | 5 | **100** |  | 0 | 1 | 4 | 1 | 1 | 0 | 0 | 5 | **100** |
|  | Foraminifera | 5 | 0 | 1 | 4 | 1 | 1 | 0 | 0 | 5 | **100** |  | 0 | 1 | 4 | 1 | 1 | 0 | 0 | 5 | **100** |
| Myzozoa | | 22 | 9 | 3 | 11 | 14 | 2 | 0 | 8 | 16 | **73** |  | 11 | 3 | 13 | 15 | 2 | 0 | 9 | 17 | **77** |
|  | Blastodiniophyceae | 2 | 1 | 0 | 1 | 1 | 0 | 0 | 0 | 1 | **50** |  | 1 | 0 | 1 | 1 | 0 | 0 | 0 | 1 | **50** |
|  | Dinophyceae | 19 | 8 | 3 | 9 | 13 | 2 | 0 | 8 | 14 | **74** |  | 10 | 3 | 11 | 14 | 2 | 0 | 9 | 15 | **79** |
|  | Noctiluciphyceae | 1 | 0 | 0 | 1 | 0 | 0 | 0 | 0 | 1 | **100** |  | 0 | 0 | 1 | 0 | 0 | 0 | 0 | 1 | **100** |
|  |  |  |  |  |  |  |  |  |  |  |  |  |  |  |  |  |  |  |  |  |  |
| Terrestrial taxa |  | 1718 | 222 | 178 | 451 | 685 | 496 | 343 | 659 | 1256 | **73** |  | 265 | 213 | 653 | 1005 | 851 | 802 | 707 | 1394 | **81** |
| Animalia | | 385 | 147 | 97 | 64 | 41 | 0 | 0 | 186 | 223 | **58** |  | 186 | 124 | 89 | 51 | 0 | 0 | 204 | 261 | **68** |
| Annelida | | 22 | 9 | 11 | 2 | 0 | 0 | 0 | 15 | 17 | **77** |  | 13 | 15 | 5 | 1 | 0 | 0 | 16 | 18 | **82** |
|  | Clitellata | 22 | 9 | 11 | 2 | 0 | 0 | 0 | 15 | 17 | **77** |  | 13 | 15 | 5 | 1 | 0 | 0 | 16 | 18 | **82** |
| Arthropoda | | 262 | 82 | 35 | 38 | 30 | 0 | 0 | 95 | 120 | **46** |  | 106 | 52 | 53 | 36 | 0 | 0 | 112 | 153 | **58** |
|  | Arachnida | 83 | 6 | 5 | 6 | 6 | 0 | 0 | 4 | 10 | **12** |  | 7 | 6 | 7 | 6 | 0 | 0 | 17 | 24 | **29** |
|  | Chilopoda | 1 | 0 | 0 | 0 | 0 | 0 | 0 | 0 | 0 | **0** |  | 0 | 0 | 0 | 0 | 0 | 0 | 0 | 0 | **0** |
|  | Collembola | 2 | 2 | 1 | 0 | 0 | 0 | 0 | 2 | 2 | **100** |  | 2 | 1 | 0 | 0 | 0 | 0 | 2 | 2 | **100** |
|  | Diplopoda | 1 | 0 | 0 | 0 | 0 | 0 | 0 | 1 | 1 | **100** |  | 1 | 0 | 0 | 0 | 0 | 0 | 1 | 1 | **100** |
|  | Insecta | 175 | 74 | 29 | 32 | 24 | 0 | 0 | 88 | 107 | **61** |  | 96 | 45 | 46 | 30 | 0 | 0 | 92 | 126 | **72** |
| Chordata | | 97 | 55 | 51 | 22 | 10 | 0 | 0 | 74 | 84 | **87** |  | 66 | 57 | 29 | 13 | 0 | 0 | 74 | 88 | **91** |
|  | Amphibia | 9 | 3 | 5 | 0 | 0 | 0 | 0 | 3 | 7 | **78** |  | 4 | 6 | 1 | 0 | 0 | 0 | 3 | 8 | **89** |
|  | Aves | 36 | 25 | 15 | 8 | 4 | 0 | 0 | 32 | 32 | **89** |  | 30 | 17 | 11 | 4 | 0 | 0 | 32 | 34 | **94** |
|  | Mammalia | 43 | 22 | 25 | 12 | 5 | 0 | 0 | 35 | 38 | **88** |  | 26 | 27 | 15 | 8 | 0 | 0 | 35 | 39 | **91** |
|  | Reptilia | 9 | 5 | 6 | 2 | 1 | 0 | 0 | 4 | 7 | **78** |  | 6 | 7 | 2 | 1 | 0 | 0 | 4 | 7 | **78** |
| Mollusca | | 2 | 0 | 0 | 0 | 0 | 0 | 0 | 0 | 0 | **0** |  | 0 | 0 | 0 | 0 | 0 | 0 | 0 | 0 | **0** |
|  | Gastropoda | 2 | 0 | 0 | 0 | 0 | 0 | 0 | 0 | 0 | **0** |  | 0 | 0 | 0 | 0 | 0 | 0 | 0 | 0 | **0** |
| Nematoda | | 1 | 0 | 0 | 1 | 1 | 0 | 0 | 1 | 1 | **100** |  | 0 | 0 | 1 | 1 | 0 | 0 | 1 | 1 | **100** |
|  | Secernentea | 1 | 0 | 0 | 1 | 1 | 0 | 0 | 1 | 1 | **100** |  | 0 | 0 | 1 | 1 | 0 | 0 | 1 | 1 | **100** |
| Platyhelminthes | | 1 | 1 | 0 | 1 | 0 | 0 | 0 | 1 | 1 | **100** |  | 1 | 0 | 1 | 0 | 0 | 0 | 1 | 1 | **100** |
|  | Turbellaria | 1 | 1 | 0 | 1 | 0 | 0 | 0 | 1 | 1 | **100** |  | 1 | 0 | 1 | 0 | 0 | 0 | 1 | 1 | **100** |
| Plantae | | 1333 | 75 | 81 | 387 | 644 | 496 | 343 | 473 | 1033 | **78** |  | 79 | 89 | 564 | 954 | 851 | 802 | 501 | 1130 | **85** |
| Tracheophyta | | 1333 | 75 | 81 | 387 | 644 | 496 | 343 | 473 | 1033 | **78** |  | 79 | 89 | 564 | 954 | 851 | 802 | 501 | 1130 | **85** |
|  | Ginkoopsida | 1 | 0 | 1 | 1 | 0 | 1 | 0 | 0 | 1 | **100** |  | 0 | 1 | 1 | 1 | 1 | 1 | 0 | 1 | **100** |
|  | Liliopsida | 288 | 15 | 22 | 63 | 126 | 106 | 52 | 131 | 217 | **75** |  | 15 | 23 | 94 | 190 | 172 | 160 | 133 | 234 | **81** |
|  | Magnoliopsida | 983 | 50 | 51 | 304 | 498 | 341 | 271 | 328 | 758 | **77** |  | 53 | 57 | 444 | 718 | 618 | 593 | 352 | 834 | **85** |
|  | Marattiopsida | 1 | 0 | 1 | 0 | 0 | 1 | 1 | 0 | 1 | **100** |  | 0 | 1 | 0 | 0 | 1 | 1 | 0 | 1 | **100** |
|  | Pinopsida | 53 | 9 | 4 | 17 | 17 | 42 | 18 | 14 | 50 | **94** |  | 10 | 4 | 23 | 39 | 53 | 43 | 16 | 53 | **100** |
|  | Polypodiopsida | 7 | 1 | 2 | 2 | 3 | 5 | 1 | 0 | 6 | **86** |  | 1 | 3 | 2 | 6 | 6 | 4 | 0 | 7 | **100** |

|  |  |  | 2016 | | | | | | | | | | | | | |
| --- | --- | --- | --- | --- | --- | --- | --- | --- | --- | --- | --- | --- | --- | --- | --- | --- |
|  |  | # of NIS | GenBank | | | | | | BOLD | | | | | | Total # | % |
|  |  |  | COI | 16S | 18S | ITS | rbcL | matK | COI | 16S | 18S | ITS | rbcL | matK |  |  |
| Aquatic taxa |  | 1383 | 719 | 569 | 600 | 355 | 242 | 94 | 623 | 6 | 5 | 75 | 192 | 91 | 1047 | **76** |
| Animalia | | 985 | 629 | 516 | 423 | 188 | 0 | 0 | 565 | 6 | 5 | 1 | 0 | 0 | 743 | **75** |
| Annelida | | 95 | 46 | 30 | 48 | 13 | 0 | 0 | 39 | 0 | 0 | 0 | 0 | 0 | 56 | **59** |
|  | Clitellata | 28 | 18 | 15 | 18 | 10 | 0 | 0 | 17 | 0 | 0 | 0 | 0 | 0 | 21 | **75** |
|  | Hirudinea | 2 | 2 | 1 | 2 | 0 | 0 | 0 | 2 | 0 | 0 | 0 | 0 | 0 | 2 | **100** |
|  | Polychaeta | 65 | 26 | 14 | 28 | 3 | 0 | 0 | 20 | 0 | 0 | 0 | 0 | 0 | 33 | **51** |
| Arthropoda | | 253 | 138 | 97 | 111 | 40 | 0 | 0 | 131 | 1 | 0 | 0 | 0 | 0 | 165 | **65** |
|  | Branchiopoda | 23 | 11 | 11 | 11 | 1 | 0 | 0 | 11 | 0 | 0 | 0 | 0 | 0 | 16 | **70** |
|  | Insecta | 17 | 9 | 3 | 6 | 4 | 0 | 0 | 9 | 0 | 0 | 0 | 0 | 0 | 10 | **59** |
|  | Malacostraca | 143 | 84 | 66 | 59 | 21 | 0 | 0 | 80 | 1 | 0 | 0 | 0 | 0 | 96 | **67** |
|  | Maxillopoda | 58 | 31 | 14 | 30 | 13 | 0 | 0 | 28 | 0 | 0 | 0 | 0 | 0 | 38 | **66** |
|  | Merostomata | 1 | 1 | 1 | 1 | 1 | 0 | 0 | 1 | 0 | 0 | 0 | 0 | 0 | 1 | **100** |
|  | Ostracoda | 8 | 0 | 0 | 2 | 0 | 0 | 0 | 0 | 0 | 0 | 0 | 0 | 0 | 2 | **25** |
|  | Pycnogonida | 3 | 2 | 2 | 2 | 0 | 0 | 0 | 2 | 0 | 0 | 0 | 0 | 0 | 2 | **67** |
| Bryozoa | | 41 | 27 | 22 | 22 | 1 | 0 | 0 | 24 | 3 | 0 | 0 | 0 | 0 | 31 | **76** |
|  | Gymnolaemata | 36 | 26 | 20 | 20 | 1 | 0 | 0 | 23 | 3 | 0 | 0 | 0 | 0 | 28 | **78** |
|  | Phylactolaemata | 2 | 1 | 2 | 1 | 0 | 0 | 0 | 1 | 0 | 0 | 0 | 0 | 0 | 2 | **100** |
|  | Not defined | 3 | 0 | 0 | 1 | 0 | 0 | 0 | 0 | 0 | 0 | 0 | 0 | 0 | 1 | **33** |
| Chordata | | 300 | 254 | 216 | 97 | 54 | 0 | 0 | 237 | 0 | 3 | 0 | 0 | 0 | 271 | **90** |
|  | Actinopterygii | 225 | 201 | 183 | 70 | 45 | 0 | 0 | 186 | 0 | 0 | 0 | 0 | 0 | 211 | **94** |
|  | Amphibia | 8 | 5 | 8 | 2 | 2 | 0 | 0 | 5 | 0 | 2 | 0 | 0 | 0 | 8 | **100** |
|  | Ascidiacea | 45 | 29 | 7 | 20 | 5 | 0 | 0 | 27 | 0 | 1 | 0 | 0 | 0 | 31 | **69** |
|  | Aves | 11 | 9 | 7 | 1 | 1 | 0 | 0 | 10 | 0 | 0 | 0 | 0 | 0 | 10 | **91** |
|  | Cephalaspidomorphi | 1 | 1 | 1 | 0 | 0 | 0 | 0 | 1 | 0 | 0 | 0 | 0 | 0 | 1 | **100** |
|  | Chondrichthyes | 3 | 3 | 3 | 0 | 1 | 0 | 0 | 3 | 0 | 0 | 0 | 0 | 0 | 3 | **100** |
|  | Mammalia | 5 | 4 | 5 | 3 | 0 | 0 | 0 | 4 | 0 | 0 | 0 | 0 | 0 | 5 | **100** |
|  | Reptilia | 2 | 2 | 2 | 1 | 0 | 0 | 0 | 1 | 0 | 0 | 0 | 0 | 0 | 2 | **100** |
| Cnidaria | | 49 | 24 | 35 | 27 | 15 | 0 | 0 | 24 | 1 | 1 | 1 | 0 | 0 | 39 | **80** |
|  | Anthozoa | 10 | 5 | 6 | 6 | 2 | 0 | 0 | 4 | 0 | 0 | 0 | 0 | 0 | 8 | **80** |
|  | Hydrozoa | 33 | 13 | 24 | 16 | 9 | 0 | 0 | 14 | 0 | 0 | 0 | 0 | 0 | 25 | **76** |
|  | Scyphozoa | 6 | 6 | 5 | 5 | 4 | 0 | 0 | 6 | 1 | 1 | 1 | 0 | 0 | 6 | **100** |
| Ctenophora | | 2 | 2 | 1 | 2 | 2 | 0 | 0 | 1 | 0 | 0 | 0 | 0 | 0 | 2 | **100** |
|  | Nuda | 1 | 1 | 0 | 1 | 1 | 0 | 0 | 0 | 0 | 0 | 0 | 0 | 0 | 1 | **100** |
|  | Tentaculata | 1 | 1 | 1 | 1 | 1 | 0 | 0 | 1 | 0 | 0 | 0 | 0 | 0 | 1 | **100** |
| Echinodermata | | 8 | 6 | 3 | 3 | 0 | 0 | 0 | 6 | 0 | 0 | 0 | 0 | 0 | 6 | **75** |
|  | Asteroidea | 5 | 4 | 3 | 3 | 0 | 0 | 0 | 4 | 0 | 0 | 0 | 0 | 0 | 4 | **80** |
|  | Holothuroidea | 1 | 1 | 0 | 0 | 0 | 0 | 0 | 1 | 0 | 0 | 0 | 0 | 0 | 1 | **100** |
|  | Ophiuroidea | 2 | 1 | 0 | 0 | 0 | 0 | 0 | 1 | 0 | 0 | 0 | 0 | 0 | 1 | **50** |
| Mollusca | | 181 | 115 | 106 | 89 | 49 | 0 | 0 | 91 | 1 | 1 | 0 | 0 | 0 | 143 | **79** |
|  | Bivalvia | 83 | 53 | 48 | 54 | 36 | 0 | 0 | 44 | 0 | 1 | 0 | 0 | 0 | 71 | **86** |
|  | Cephalopoda | 2 | 2 | 2 | 0 | 0 | 0 | 0 | 1 | 0 | 0 | 0 | 0 | 0 | 2 | **100** |
|  | Gastropoda | 96 | 60 | 56 | 35 | 13 | 0 | 0 | 46 | 1 | 0 | 0 | 0 | 0 | 70 | **73** |
| Myxozoa | | 2 | 0 | 0 | 1 | 1 | 0 | 0 | 0 | 0 | 0 | 0 | 0 | 0 | 1 | **50** |
|  | Myxosporea | 2 | 0 | 0 | 1 | 1 | 0 | 0 | 0 | 0 | 0 | 0 | 0 | 0 | 1 | **50** |
| Nematoda | | 3 | 1 | 1 | 1 | 1 | 0 | 0 | 2 | 0 | 0 | 0 | 0 | 0 | 2 | **67** |
|  | Secernentea | 3 | 1 | 1 | 1 | 1 | 0 | 0 | 2 | 0 | 0 | 0 | 0 | 0 | 2 | **67** |
| Platyhelminthes | | 25 | 9 | 1 | 10 | 10 | 0 | 0 | 5 | 0 | 0 | 0 | 0 | 0 | 15 | **60** |
|  | Cestoidea | 7 | 5 | 0 | 3 | 2 | 0 | 0 | 2 | 0 | 0 | 0 | 0 | 0 | 5 | **71** |
|  | Monogenea | 7 | 2 | 1 | 5 | 6 | 0 | 0 | 2 | 0 | 0 | 0 | 0 | 0 | 7 | **100** |
|  | Trematoda | 6 | 2 | 0 | 2 | 2 | 0 | 0 | 1 | 0 | 0 | 0 | 0 | 0 | 3 | **50** |
|  | Turbellaria | 5 | 0 | 0 | 0 | 0 | 0 | 0 | 0 | 0 | 0 | 0 | 0 | 0 | 0 | **0** |
| Porifera | | 19 | 7 | 4 | 11 | 2 | 0 | 0 | 5 | 0 | 0 | 0 | 0 | 0 | 11 | **58** |
|  | Demospongiae | 19 | 7 | 4 | 11 | 2 | 0 | 0 | 5 | 0 | 0 | 0 | 0 | 0 | 11 | **58** |
| Rotifera | | 7 | 0 | 0 | 1 | 0 | 0 | 0 | 0 | 0 | 0 | 0 | 0 | 0 | 1 | **14** |
|  | Monogononta | 7 | 0 | 0 | 1 | 0 | 0 | 0 | 0 | 0 | 0 | 0 | 0 | 0 | 1 | **14** |
| Chromista | | 75 | 21 | 19 | 34 | 25 | 41 | 0 | 16 | 0 | 0 | 7 | 29 | 2 | 56 | **75** |
| Haptophyta | | 2 | 0 | 1 | 1 | 1 | 0 | 0 | 0 | 0 | 0 | 0 | 1 | 0 | 1 | **50** |
|  | Prymnesiophyceae | 2 | 0 | 1 | 1 | 1 | 0 | 0 | 0 | 0 | 0 | 0 | 1 | 0 | 1 | **50** |
| Labyrinthulomycota | | 1 | 0 | 0 | 1 | 1 | 0 | 0 | 0 | 0 | 0 | 0 | 0 | 0 | 1 | **100** |
|  | Labyrinthulomycetes | 1 | 0 | 0 | 1 | 1 | 0 | 0 | 0 | 0 | 0 | 0 | 0 | 0 | 1 | **100** |
| Ochrophyta | | 72 | 21 | 18 | 32 | 23 | 41 | 0 | 16 | 0 | 0 | 7 | 28 | 2 | 54 | **75** |
|  | Bacillariophyceae | 7 | 0 | 1 | 4 | 3 | 2 | 0 | 0 | 0 | 0 | 1 | 0 | 0 | 5 | **71** |
|  | Chrysophyceae | 1 | 0 | 0 | 0 | 0 | 0 | 0 | 0 | 0 | 0 | 0 | 0 | 0 | 0 | **0** |
|  | Coscinodiscophyceae | 21 | 4 | 6 | 13 | 8 | 13 | 0 | 4 | 0 | 0 | 5 | 8 | 2 | 16 | **76** |
|  | Pelagophyceae | 1 | 0 | 1 | 1 | 1 | 1 | 0 | 0 | 0 | 0 | 0 | 0 | 0 | 1 | **100** |
|  | Phaeophyceae | 38 | 16 | 7 | 12 | 9 | 23 | 0 | 11 | 0 | 0 | 1 | 19 | 0 | 29 | **76** |
|  | Raphidophyceae | 3 | 1 | 3 | 2 | 2 | 2 | 0 | 1 | 0 | 0 | 0 | 1 | 0 | 3 | **100** |
|  | Xanthophyceae | 1 | 0 | 0 | 0 | 0 | 0 | 0 | 0 | 0 | 0 | 0 | 0 | 0 | 0 | **0** |
| Plantae | | 285 | 55 | 30 | 123 | 126 | 197 | 93 | 32 | 0 | 0 | 61 | 163 | 89 | 224 | **79** |
| Charophyta | | 2 | 0 | 1 | 2 | 0 | 2 | 2 | 0 | 0 | 0 | 0 | 2 | 2 | 2 | **100** |
|  | Charophyceae | 2 | 0 | 1 | 2 | 0 | 2 | 2 | 0 | 0 | 0 | 0 | 2 | 2 | 2 | **100** |
| Chlorophyta | | 44 | 1 | 7 | 15 | 14 | 23 | 0 | 3 | 0 | 0 | 7 | 14 | 0 | 27 | **61** |
|  | Bryopsidophyceae | 13 | 0 | 5 | 4 | 4 | 10 | 0 | 2 | 0 | 0 | 2 | 6 | 0 | 10 | **77** |
|  | Trebouxiophyceae | 1 | 0 | 1 | 1 | 0 | 1 | 0 | 0 | 0 | 0 | 0 | 1 | 0 | 1 | **100** |
|  | Ulvophyceae | 30 | 1 | 1 | 10 | 10 | 12 | 0 | 1 | 0 | 0 | 5 | 7 | 0 | 16 | **53** |
| Marchantiophyta | | 1 | 0 | 0 | 0 | 0 | 0 | 0 | 0 | 0 | 0 | 0 | 0 | 0 | 0 | **0** |
|  | Marchantiopsida | 1 | 0 | 0 | 0 | 0 | 0 | 0 | 0 | 0 | 0 | 0 | 0 | 0 | 0 | **0** |
| Rodophyta | | 114 | 47 | 8 | 38 | 23 | 71 | 2 | 24 | 0 | 0 | 1 | 48 | 0 | 84 | **74** |
|  | Bangiophyceae | 1 | 1 | 0 | 1 | 1 | 1 | 0 | 0 | 0 | 0 | 0 | 0 | 0 | 1 | **100** |
|  | Compsopogonophyceae | 1 | 0 | 0 | 0 | 0 | 0 | 0 | 0 | 0 | 0 | 0 | 0 | 0 | 0 | **0** |
|  | Florideophyceae | 110 | 45 | 6 | 35 | 21 | 68 | 2 | 24 | 0 | 0 | 1 | 47 | 0 | 81 | **74** |
|  | Rhodellophyceae | 2 | 1 | 2 | 2 | 1 | 2 | 0 | 0 | 0 | 0 | 0 | 1 | 0 | 2 | **100** |
| Tracheophyta | | 124 | 7 | 14 | 68 | 89 | 101 | 89 | 5 | 0 | 0 | 53 | 99 | 87 | 111 | **90** |
|  | Liliopsida | 57 | 3 | 5 | 38 | 45 | 48 | 46 | 2 | 0 | 0 | 23 | 46 | 43 | 53 | **93** |
|  | Magnoliopsida | 62 | 4 | 9 | 27 | 41 | 48 | 42 | 3 | 0 | 0 | 28 | 48 | 43 | 53 | **85** |
|  | Polypodiopsida | 5 | 0 | 0 | 3 | 3 | 5 | 1 | 0 | 0 | 0 | 2 | 5 | 1 | 5 | **100** |
| Protozoa | | 38 | 14 | 4 | 20 | 16 | 4 | 1 | 10 | 0 | 0 | 6 | 0 | 0 | 24 | **63** |
| Cercozoa | | 4 | 0 | 1 | 0 | 0 | 0 | 0 | 0 | 0 | 0 | 0 | 0 | 0 | 1 | **25** |
|  | Ascetosporea | 1 | 0 | 1 | 0 | 0 | 0 | 0 | 0 | 0 | 0 | 0 | 0 | 0 | 1 | **100** |
|  | Gromiidea | 3 | 0 | 0 | 0 | 0 | 0 | 0 | 0 | 0 | 0 | 0 | 0 | 0 | 0 | **0** |
| Ciliophora | | 6 | 0 | 0 | 1 | 0 | 1 | 1 | 0 | 0 | 0 | 0 | 0 | 0 | 1 | **17** |
|  | Ciliatea | 2 | 0 | 0 | 0 | 0 | 0 | 0 | 0 | 0 | 0 | 0 | 0 | 0 | 0 | **0** |
|  | Kinetofragminophora | 1 | 0 | 0 | 1 | 0 | 1 | 1 | 0 | 0 | 0 | 0 | 0 | 0 | 1 | **100** |
|  | Oligohymenophorea | 3 | 0 | 0 | 0 | 0 | 0 | 0 | 0 | 0 | 0 | 0 | 0 | 0 | 0 | **0** |
| Euglenozoa | | 1 | 0 | 0 | 0 | 0 | 0 | 0 | 0 | 0 | 0 | 0 | 0 | 0 | 0 | **0** |
|  | Kinetoplastea | 1 | 0 | 0 | 0 | 0 | 0 | 0 | 0 | 0 | 0 | 0 | 0 | 0 | 0 | **0** |
| Foraminifera | | 5 | 1 | 1 | 4 | 1 | 1 | 0 | 0 | 0 | 0 | 0 | 0 | 0 | 5 | **100** |
|  | Foraminifera | 5 | 1 | 1 | 4 | 1 | 1 | 0 | 0 | 0 | 0 | 0 | 0 | 0 | 5 | **100** |
| Myzozoa | | 22 | 13 | 2 | 15 | 15 | 2 | 0 | 10 | 0 | 0 | 6 | 0 | 0 | 17 | **77** |
|  | Blastodiniophyceae | 2 | 2 | 1 | 2 | 2 | 0 | 0 | 2 | 0 | 0 | 0 | 0 | 0 | 2 | **100** |
|  | Dinophyceae | 19 | 11 | 1 | 12 | 13 | 2 | 0 | 8 | 0 | 0 | 6 | 0 | 0 | 14 | **74** |
|  | Noctiluciphyceae | 1 | 0 | 0 | 1 | 0 | 0 | 0 | 0 | 0 | 0 | 0 | 0 | 0 | 1 | **100** |
|  |  |  |  |  |  |  |  |  |  |  |  |  |  |  |  |  |
| Terrestrial taxa |  | 1718 | 266 | 360 | 847 | 1101 | 1008 | 999 | 284 | 8 | 2 | 639 | 936 | 887 | 1460 | **85** |
| Animalia | | 385 | 230 | 148 | 117 | 62 | 3 | 3 | 235 | 8 | 1 | 3 | 0 | 0 | 286 | **74** |
| Annelida | | 22 | 15 | 15 | 8 | 1 | 0 | 0 | 16 | 0 | 0 | 0 | 0 | 0 | 18 | **82** |
|  | Clitellata | 22 | 15 | 15 | 8 | 1 | 0 | 0 | 16 | 0 | 0 | 0 | 0 | 0 | 18 | **82** |
| Arthropoda | | 262 | 135 | 58 | 76 | 51 | 2 | 2 | 133 | 1 | 1 | 3 | 0 | 0 | 176 | **67** |
|  | Arachnida | 83 | 15 | 8 | 14 | 6 | 1 | 1 | 15 | 0 | 0 | 0 | 0 | 0 | 36 | **43** |
|  | Chilopoda | 1 | 1 | 1 | 1 | 0 | 0 | 0 | 1 | 0 | 0 | 0 | 0 | 0 | 1 | **100** |
|  | Collembola | 2 | 2 | 0 | 0 | 0 | 0 | 0 | 2 | 0 | 0 | 0 | 0 | 0 | 2 | **100** |
|  | Diplopoda | 1 | 1 | 0 | 0 | 0 | 0 | 0 | 1 | 0 | 0 | 0 | 0 | 0 | 1 | **100** |
|  | Insecta | 175 | 116 | 49 | 61 | 45 | 1 | 1 | 114 | 1 | 1 | 3 | 0 | 0 | 136 | **78** |
| Chordata | | 97 | 78 | 75 | 31 | 9 | 1 | 1 | 83 | 7 | 0 | 0 | 0 | 0 | 89 | **92** |
|  | Amphibia | 9 | 6 | 8 | 1 | 0 | 0 | 0 | 7 | 5 | 0 | 0 | 0 | 0 | 8 | **89** |
|  | Aves | 36 | 32 | 23 | 10 | 3 | 1 | 1 | 33 | 0 | 0 | 0 | 0 | 0 | 34 | **94** |
|  | Mammalia | 43 | 35 | 37 | 19 | 5 | 0 | 0 | 39 | 2 | 0 | 0 | 0 | 0 | 40 | **93** |
|  | Reptilia | 9 | 5 | 7 | 1 | 1 | 0 | 0 | 4 | 0 | 0 | 0 | 0 | 0 | 7 | **78** |
| Mollusca | | 2 | 1 | 0 | 0 | 0 | 0 | 0 | 1 | 0 | 0 | 0 | 0 | 0 | 1 | **50** |
|  | Gastropoda | 2 | 1 | 0 | 0 | 0 | 0 | 0 | 1 | 0 | 0 | 0 | 0 | 0 | 1 | **50** |
| Nematoda | | 1 | 0 | 0 | 1 | 1 | 0 | 0 | 1 | 0 | 0 | 0 | 0 | 0 | 1 | **100** |
|  | Secernentea | 1 | 0 | 0 | 1 | 1 | 0 | 0 | 1 | 0 | 0 | 0 | 0 | 0 | 1 | **100** |
| Platyhelminthes | | 1 | 1 | 0 | 1 | 0 | 0 | 0 | 1 | 0 | 0 | 0 | 0 | 0 | 1 | **100** |
|  | Turbellaria | 1 | 1 | 0 | 1 | 0 | 0 | 0 | 1 | 0 | 0 | 0 | 0 | 0 | 1 | **100** |
| Plantae | | 1333 | 36 | 212 | 730 | 1039 | 1005 | 996 | 49 | 0 | 1 | 636 | 936 | 887 | 1174 | **88** |
| Tracheophyta | | 1333 | 36 | 212 | 730 | 1039 | 1005 | 996 | 49 | 0 | 1 | 636 | 936 | 887 | 1174 | **88** |
|  | Ginkoopsida | 1 | 0 | 1 | 1 | 1 | 1 | 1 | 0 | 0 | 0 | 1 | 1 | 1 | 1 | **100** |
|  | Liliopsida | 288 | 4 | 42 | 131 | 209 | 205 | 207 | 15 | 0 | 0 | 113 | 190 | 173 | 249 | **86** |
|  | Magnoliopsida | 983 | 27 | 149 | 564 | 782 | 739 | 735 | 30 | 0 | 1 | 488 | 688 | 664 | 863 | **88** |
|  | Marattiopsida | 1 | 0 | 1 | 1 | 0 | 1 | 1 | 0 | 0 | 0 | 1 | 1 | 1 | 1 | **100** |
|  | Pinopsida | 53 | 5 | 18 | 29 | 42 | 53 | 46 | 4 | 0 | 0 | 30 | 50 | 44 | 53 | **100** |
|  | Polypodiopsida | 7 | 0 | 1 | 4 | 5 | 6 | 6 | 0 | 0 | 0 | 3 | 6 | 4 | 7 | **100** |

**APPENDIX 3** Number of non-indigenous species (NIS) per class for the three most species-abundant aquatic Animalia and three aquatic Plantae phyla, and number of NIS with at least one sequence in at least one genetic database in 2010, 2012 and 2016. Percentage cover for 2010, 2012 and 2016 are shown in brackets, respectively.


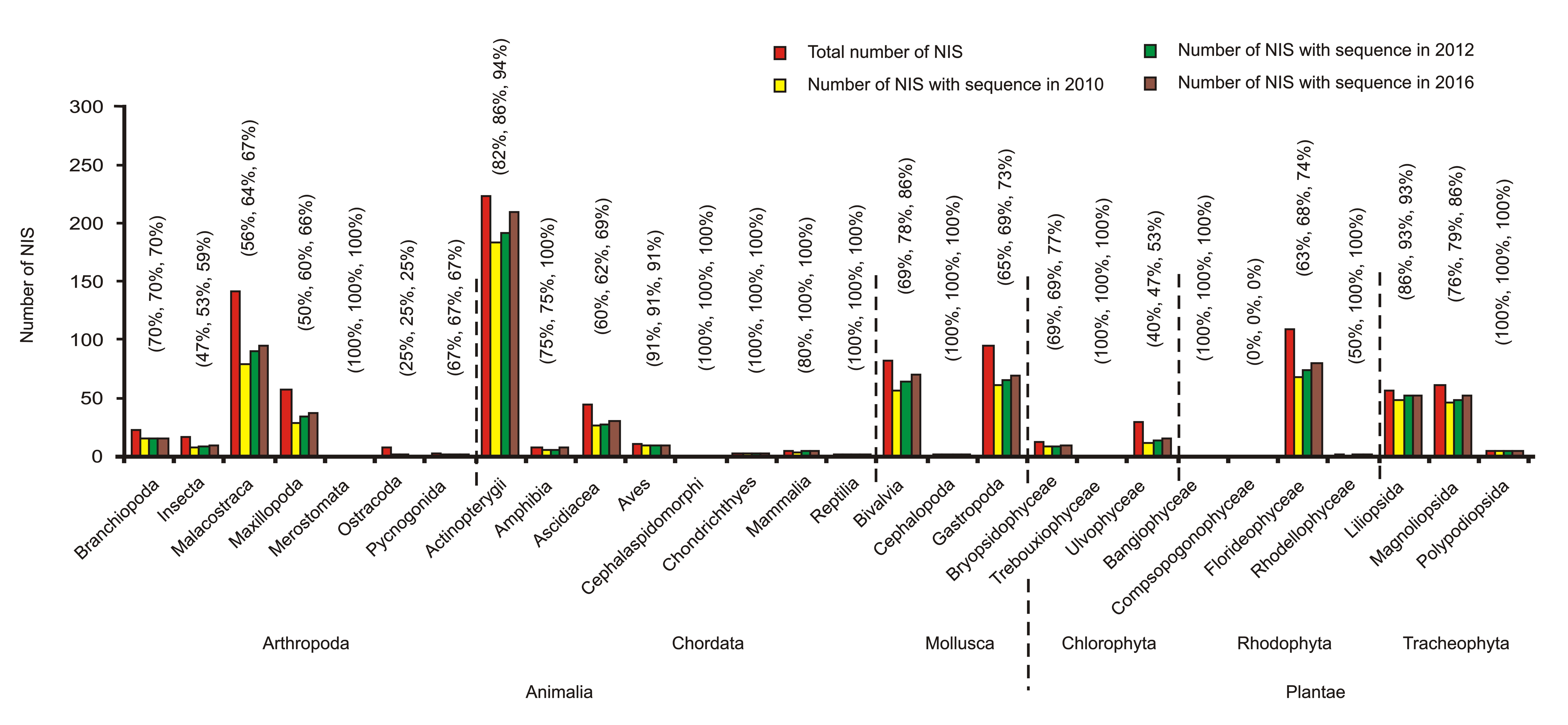


**APPENDIX 4** Number of non-indigenous species (NIS) per class for the two most species-abundant terrestrial Animalia and one terrestrial Plantae phyla, and number of NIS with at least one sequence in at least one genetic database in 2010, 2012 and 2016. Percentage cover for 2010, 2012 and 2016 are shown in brackets, respectively.


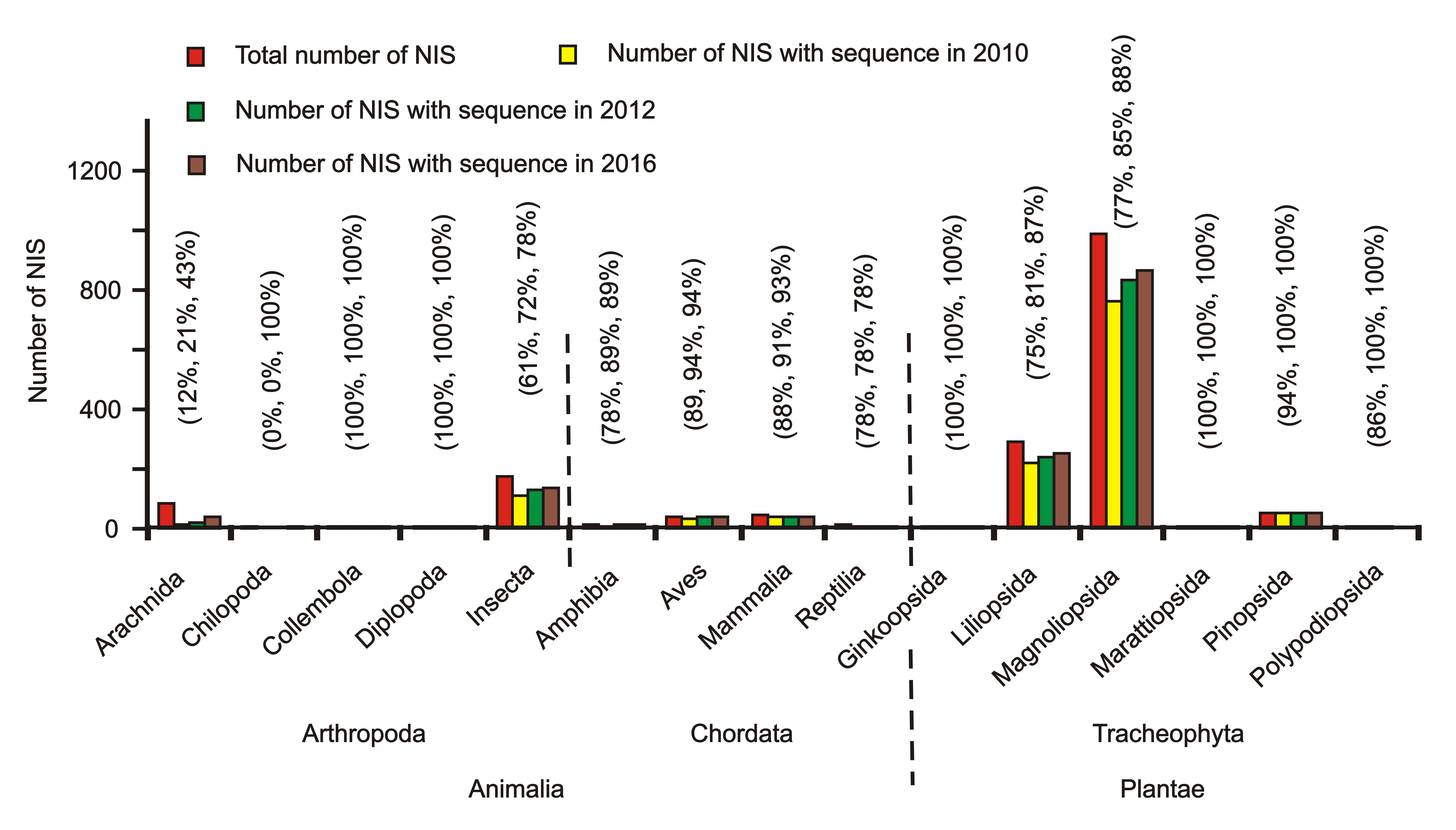

Supplement: Supplementary file 1 — Supplementary material 1 (DOC 4556 kb) [file 10530_2016_1134_MOESM1_ESM.doc]
